# Apical-basal polarity precisely determines intestinal stem cell number by regulating Prospero threshold

**DOI:** 10.1016/j.celrep.2023.112093

**Published:** 2023-02-10

**Authors:** Song Wu, Yang Yang, Ruizhi Tang, Song Zhang, Peizhong Qin, Rong Lin, Neus Rafel, Elena M. Lucchetta, Benjamin Ohlstein, Zheng Guo

**Affiliations:** 1 Department of Medical Genetics, School of Basic Medicine, Institute for Brain Research, Tongji Medical College, Huazhong University of Science and Technology, Wuhan 430022, China; 2 Division of Gastroenterology, Union Hospital, Tongji Medical College, Huazhong University of Science and Technology, Wuhan 430022, China; 3 Department of Genetics and Development, Columbia University Medical Center, New York, NY 10032, USA; 4 Cell Architecture Research Center, Huazhong University of Science and Technology, Wuhan, Hubei 430030, China; 5 Present address: Division of Gastroenterology, the Central Hospital of Wuhan, Tongji Medical College, Huazhong University of Science and Technology, Wuhan 430014, China; 6 Present address: Department of Medical Laboratory, the Central Hospital of Wuhan, Tongji Medical College, Huazhong University of Science and Technology, Wuhan 430014, China; 7 Present address: Children’s Research Institute and Department of Pediatrics, University of Texas Southwestern Medical Center, Dallas, TX, USA; 8 These authors contributed equally; 9 Lead contact

## Abstract

Apical-basal polarity and cell-fate determinants are crucial for the cell fate and control of stem cell numbers. However, their interplay leading to a precise stem cell number remains unclear. *Drosophila* pupal intestinal stem cells (pISCs) asymmetrically divide, generating one apical ISC progenitor and one basal Prospero (Pros)^+^ enteroendocrine mother cell (EMC), followed by symmetric divisions of each daughter before adulthood, providing an ideal system to investigate the outcomes of polarity loss. Using lineage tracing and *ex vivo* live imaging, we identify an interlocked polarity regulation network precisely determining ISC number: Bazooka inhibits Pros accumulation by activating Notch signaling to maintain stem cell fate in pISC apical daughters. A threshold of Pros promotes differentiation to EMCs and avoids ISC-like cell fate, and over-threshold of Pros inhibits *miranda* expression to ensure symmetric divisions in pISC basal daughters. Our work suggests that a polarity-dependent threshold of a differentiation factor precisely controls stem cell number.

## In brief

By studying *Drosophila* pupal intestinal stem cells, Wu et al. find that the number of stem cells is precisely determined by interlocked polarity components via control of Prospero threshold. Thus, the final number of stem cells in a developing tissue is predetermined by internal gene-regulatory networks of stem cells.

## Graphical Abstract

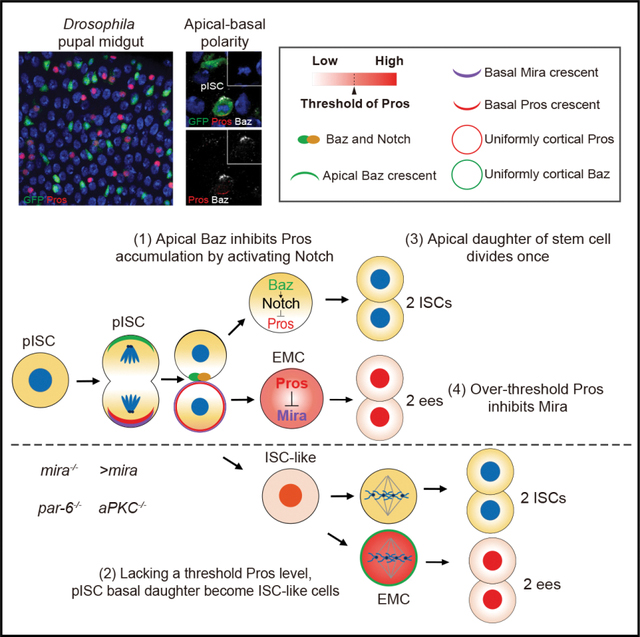

## INTRODUCTION

The manner in which the number of adult stem cells is determined during tissue development has been an intriguing question for a long time. Mammalian hematopoietic stem cells and *Drosophila* germline stem cells, for example, are maintained by niches, which are supportive microenvironments.^1–3^ How the number of epithelial stem cells, which lack prominent niche structures, is controlled is largely unknown. Recently, mammalian intestine subepithelial telocytes, which secrete canonical Wnt molecules to sustain a gradient from the bottom of the crypt to the tip of the villus to maintain the stemness of the crypt Lgr5^+^ epithelial stem cells, were confirmed as mammalian intestinal stem cell (ISC) niche cells.^4^ However, the intestinal niche theory cannot explain the reason for the presence of approximately 3.5 Lgr5^+^ ISCs per sectioned crypt;^5^ thus, an additional mechanism independent from Wnt signaling must be involved in the regulation of the ISC number.

Polarity and asymmetric divisions are used by *Drosophila* embryonic and larval neuroblasts (NBs) to maintain stem cell number and guarantee a proper differentiation of daughter cells.^6–9^ Embryonic NBs divide asymmetrically along the apical-basal axis to produce a large self-renewing apical NB and a small basal ganglion mother cell (GMC).^9^ *Bazooka* (*Baz*, the homolog of *Par-3*) physically interacts with Par-6 and atypical protein kinase C (aPKC), forming the Par complex, localized on the apical cell membrane of mitotic NB that establishes an apical cue for NB asymmetric division.^10–12^ aPKC phosphorylates Miranda (Mira) to form a Mira crescent that basally localizes during NB mitotic division.^13,14^ Mira functions as an adaptor protein that binds the homeodomain-containing transcription factor Prospero (Pros, ortholog of mammalian Prox1) to ensure that the basal sibling faithfully differentiates into a GMC that generates two post-mitotic neurons or glial cells.^15–18^ Besides Mira, aPKC also phosphorylates Numb to direct it on the basal cortex of mitotic NBs.^19^ Numb facilitates Notch receptor endocytosis, leading to the inhibition of Notch signaling in the GMC.^20^ Defects in Pros, Numb, and Mira cause basal daughter de-differentiation into NB-like cells and tumor formation in *Drosophila* larva brain.^21,22^ In addition to NB, *Drosophila* sensory organ precursors (SOPs) also undergo asymmetric divisions that create a different cell fate among daughter cells by establishing uneven Notch activities between them.^23–25^ Also, the Par complex directs asymmetric divisions in adult ISCs,^26^ and spindle orientation regulates adult ISC fate.^27^ However, it is not yet known whether adult ISC number is determined by cell polarity during intestinal development.

**Figure 1.**
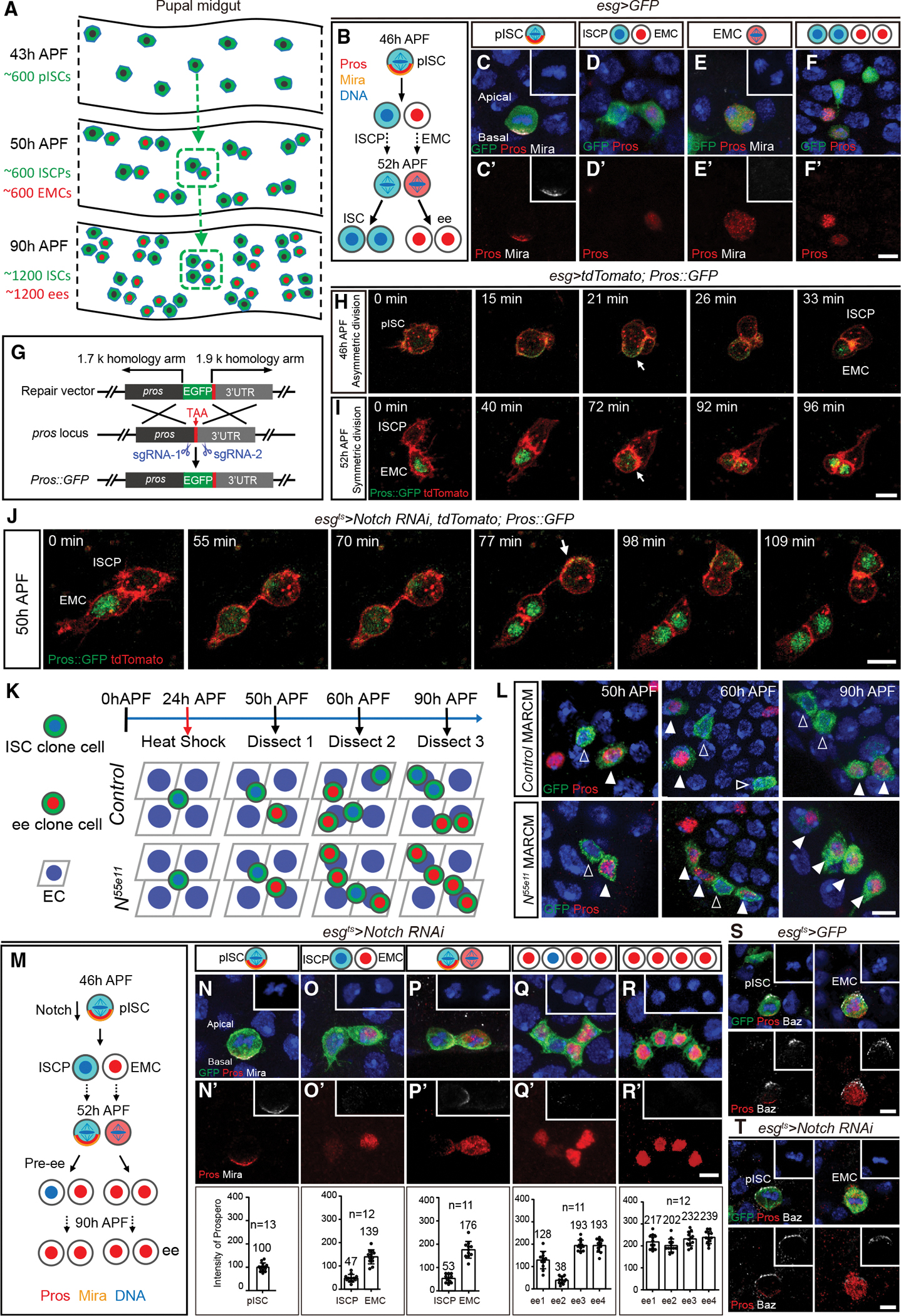
Asymmetrical division of *Notch*-deficient pISC and *Notch* signaling requirement for inhibiting Pros expression in the apical daughter (A) Schematic representation of increasing ISC number during pupal intestinal development. (B) The division pattern of pISC lineage. (C) Corresponding to schematic in (B), Mira (white) and Pros (red) asymmetrically localize on the basal cortex during pISC mitosis. Here and in all other images, blue indicates 4′,6-diamidino-2-phenylindole (DAPI), red indicates Pros, and green indicates GFP except where otherwise specified. (D) One ISCP and one EMC produced by the asymmetric division of pISC. (E) Mira absence during the symmetric division of EMC. (F) Symmetric division of the ISCP and EMC resulting in a pair of ISCs and a pair of ee cells, respectively. (G) EGFP knockin strategy at the 3′ end of *pros* by CRISPR-Cas9. (H and I) Single frames from time-lapse movies of a pISC undergoing an asymmetric division (H) and an EMC undergoing symmetric division (I). Red, *tdTomato*; green, Pros:GFP. Arrow in (H) indicates basal localization of Pros:GFP; arrow in (I) indicates diffuse cytoplasmic localization of Pros:GFP. See also Videos S1 and S2. (J) Single frames from time-lapse movies of an EMC undergoing symmetric division followed by an ISCP undergoing asymmetric division in *Notch* knockdown pupal intestines. Arrow indicates asymmetric localization of Pros:GFP. See also Video S3. (K and L) Schematic representation (K) and representative samples (L) of control and *N*^*55e11*^ MARCM clones induced at 24 h APF and examined at 50 h, 60 h, and 90 h APF. GFP, clone cells. Here and in all other images, the empty arrowhead points to a Pros^−^ cell while the white arrowhead points to a Pros^+^ cell. (M) Schematic representation of the *Notch* knockdown pISC lineage. (N) Mira and Pros are co-localized on the basal membrane of dividing pISCs. (O) Low level of Pros expression in the ISCP. (P) Mira and Pros are co-localized as a crescent on the ISCP, and EMC divides symmetrically. (Q) Three Pros^+^ and one weak Pros^+^ cell cluster. (R) Four ee cell clusters prior to eclosion. (S and T) Apical Baz crescent staining at metaphase of pISC asymmetric division and EMC symmetric division in *esg*^*ts*^*>GFP* (S) and *esg*^*ts*^*>Notch RNAi* (T) midguts. Insets show DAPI (blue) and Baz (white) staining. In (N) to (R), quantification of the intensity of Pros staining at each stage is shown below the images. The average intensity of Pros staining (AIPS) at metaphase of pISC is considered as 100. ee2 in (Q) was the pre-ee, which had the weakest Pros staining. The number above the error bar indicates the AIPS value. n denotes number of measured samples. Data are presented as mean ± SD. Scale bars, 5 μm.

The development of *Drosophila* pupal midgut is an excellent model for studying the mechanisms of ISC number regulation. Unlike *Drosophila* adult midgut ISCs which give rise to either absorptive enterocytes, hormone-producing enteroendocrine (ee) cells, or ISCs,^28–31^ pupal ISCs (pISCs) give rise to ee cells or ISCs.^32^ Midway during pupal development, at 43 h after pupal formation (APF), approximately 600 *escargot* (*esg*)^+^ pISCs in the pupal midgut are present.^32,33^ From this time point onward, pISCs start to express Pros and subsequently undergo apical-basal oriented asymmetric divisions, generating an apical ISC progenitor (ISCP) and one basal Pros^+^ ee mother cell (EMC). EMC expresses the Notch signaling ligand Delta, activating the Notch pathway in the ISCP to maintain the stem cell fate.^32^ Each ISCP and EMC will then symmetrically divide to form a pair of ISCs and a pair of ee cells, resulting in ~1,200 ISCs and ~1,200 ee cells in the pupal intestine before eclosion (Figures 1A–1F).^32^ Using this reliable system to unambiguously track ISC number, we identified an interplay among apical-basal polarity components and determined that the segregation of Pros precisely controls stem cell number and cell fate.

## RESULTS

### *Notch*-deficient pISC divides asymmetrically, and Notch signaling is required to inhibit Pros expression in the apical daughter

To better understand asymmetric division of pISCs and the dynamic localization of the key cell-fate determinant Pros, we generated, by CRISPR-Cas9 editing,^34^ in-frame knockin of a GFP tag at the 3′ end of the *pros* coding sequence (Figures 1G and S1A). Pros:GFP was then combined with ISC lineage driver *esg-Gal4*^30^ driving *UAS-tdTomato*, and a pupal midgut *ex vivo* live-imaging platform was set up to enable the performance of time-lapse imaging (Figure S1B and STAR Methods). Consistent with the immunostaining results of fixed tissues (Figures 1B–1F), our movies revealed that Pros:GFP formed a basal crescent in mitotic pISCs at approximately 46 h APF and was asymmetrically segregated to the EMC nucleus after cytokinesis (Figure 1H and Video S1). At approximately 52 h APF, Pros:GFP was ubiquitously distributed in dividing EMCs, resulting in the production of two ee cells (Figure 1I and Video S2). These results validate the use of our imaging platform to explore the *in vivo* situation of the pISC lineage.

Defects in Notch signaling result in four ee cells in one pISC lineage before eclosion.^32^ However, how these four ee cells develop has not been investigated. To answer this question, the temperature-inducible progenitor cell driver *esg-Gal4 tub-Gal80*^*ts*^ (*esg*^*ts*^)^30^ was used to drive RNA interference (RNAi) against *Notch* in the pISC lineage. Animals were transferred to the permissive temperature (30°C) from white pupae (0 h APF), and time-lapse movies were performed at 50 h APF. Our movie revealed asymmetric segregation of Pros during ISCP mitosis and symmetric segregation of Pros in EMC in *Notch* knockdown pISC lineage (Figure 1J and Video S3), suggesting that *Notch*-deficient pISCs give rise to one EMC and one ISCP, after which the ISCP asymmetrically divides before making two ee cells. To confirm our time-lapse result, control and *Notch*^*55e11*^ null mosaic analysis with repressible cell marker (MARCM) clones^35^ were induced at 24 h APF, and the cellular composition of the clones was quantified at 50 h, 60 h, and 90 h APF (Figure 1K). Homozygous clones became positively labeled with GFP after flippase-catalyzed recombination. Both *control* (n = 47) and *Notch*^*55e11*^ (n = 65) 2-cell clones contained one Pros^+^ cell and one Pros^−^ cell at 50 h APF. While all 4-cell control clones contained two Pros^+^ cells and two Pros^−^ cells at 60 h (n = 32) and 90 h (n = 47) APF, 71% of the 4-cell *Notch*^*55e11*^ clones (n = 79) contained three Pros^+^ cells and one Pros^−^ cell at 60 h APF, and 100% of 4-cell *Notch*^*55e11*^ MARCM clones (n = 91) contained four ee cells at 90 h APF (Figure 1L). Our results suggest that *Notch* mutant pISC first divides asymmetrically just like the wild type, generating one EMC and one ISCP, after which the mutant ISCP divides asymmetrically, giving rise to a Pros^+^ cell and a Pros^−^ cell. Finally, the Pros^−^ cell becomes a Pros^+^ cell before eclosion (Figure 1M).

To explore the mechanism of *Notch* mutant lineage formation, Pros adaptor protein Mira was examined in *esg*^*ts*^>*Notch RNAi* flies. The Mira-Pros basal crescent was present in *Notch* silenced pISCs during asymmetric divisions (Figures 1N and S1E1), then Pros and Mira were expressed in *Notch* knockdown ISCPs (Figures 1O and S1E2) and underwent the next round of asymmetric division while EMCs simultaneously underwent symmetric division (Figures 1P and S1E3). Later, Pros was continuously expressed in pre-ee cells (Figures 1Q and S1E4), becoming the fourth ee cell in Notch silenced pISC lineage (Figures 1R and S1E5). The above results suggest that Notch signaling is required for repressing Pros expression in the apical daughter. In addition, Par complex components Baz and aPKC localized on the apical cortex of *control* and *Notch* knockdown pISCs and EMCs during mitotic divisions (Figures 1S, 1T, and S1F–S1I) explained Mira and Pros localization during mitosis. Furthermore, spindle orientation during metaphase of pISCs and EMCs in *control* and *Notch RNAi* pupal guts was evaluated by measuring the angle formed between the basal cell membrane and the dividing spindles marked by α-tubulin. There were no differences in division angles between *control* and Notch knockdown during asymmetric divisions (Figure S1J) or symmetric divisions (Figure S1K). Together, our results demonstrate that Notch signaling is not required for the establishment of apical-basal polarity but is required for inhibiting Pros expression in ISCPs.

We next assessed how Notch regulates Pros expression in the pISC lineage. *pros* transcriptional indicator *pros-LacZ*^36^ staining was dramatically increased in *Notch* knockdown pupal intestines (Figures S1L–S1N), indicating Notch repressed Pros expression through the transcriptional level. To investigate the Notch pathway mechanism by which *pros* transcription is regulated, we carried out a whole-genome RNAi screen in pISC lineage. From the screen, we found that knockdown *phyllopod* (*phyl*), a negative regulator of transcriptional repressor Tramtrack69 (Ttk69),^37–39^ blocked pISCs differentiation into Pros^+^ ee cells (Figure S1O). Simultaneously, knockdown *Notch* and *phly* resembled the *phyl* knockdown phenotype (Figure S1O), suggesting that Notch pathway inhibits *pros* transcription through inhibiting Phyl. Since the Phyl-Ttk69-Acheate-Scute Complex-*pros* transcriptional regulation axis is well established in adult ISC lineage,^40–46^ we conclude that Notch is required to repress *pros* transcription by inhibiting Phyl expression in the ISCP.

How can we ensure that the Notch signaling is activated in apical daughter after pISC asymmetric division? Notch asymmetry in *Drosophila* NB, SOP, and adult ISC lineages is regulated by Numb.^47–49^ Using Numb antibody^24^ and a functional GFP-tagged Numb (Numb:GFP),^50^ basal cortical enriched Numb was observed in dividing pISCs (Figures S2A and S2B). However, no difference was observed between *numb* null mutants and *control* MARCM clones (Figures S2C and S2D). Consistent with the mutant clone results, knockdown *numb* did not change Mira/Pros localization, Baz distribution, or the spindle orientations during pISC mitotic divisions (Figures S2E–S2G). Notably, Notch signaling reporter *NRE*-lacZ^51^ staining was positive in ISCPs and negative in EMCs in *numb* knockdown animals (Figures S2H and S2I), suggesting that Numb is dispensable for Notch asymmetry formation, leading us to consider an alternative mechanism.

### Ectopic Mira expression converts the pISC basal daughter to an ISC-like cell

Apical-basal polarity exists both in *wild-type* and *Notch* knockdown EMCs represented by the apical Baz and aPKC localization (Figures 1S, 1T, S1F, and S1G). We wondered why the EMC did not undergo asymmetric divisions by behaving like a pISC. Based on the observations that Mira was absent in EMCs (Figures 1E and 1P), we hypothesized that Mira acted as a key factor to distinguish pISCs from EMCs. In such a scenario, ectopic expression of Mira in the pISC lineage could force the EMCs to enter the asymmetric divisions, behaving like a pISC (we term this cell an ISC-like cell), and continuously generate apical ISCPs and ISC-like cells, forming an ISC tumor in pupal midgut.

**Figure 2.**
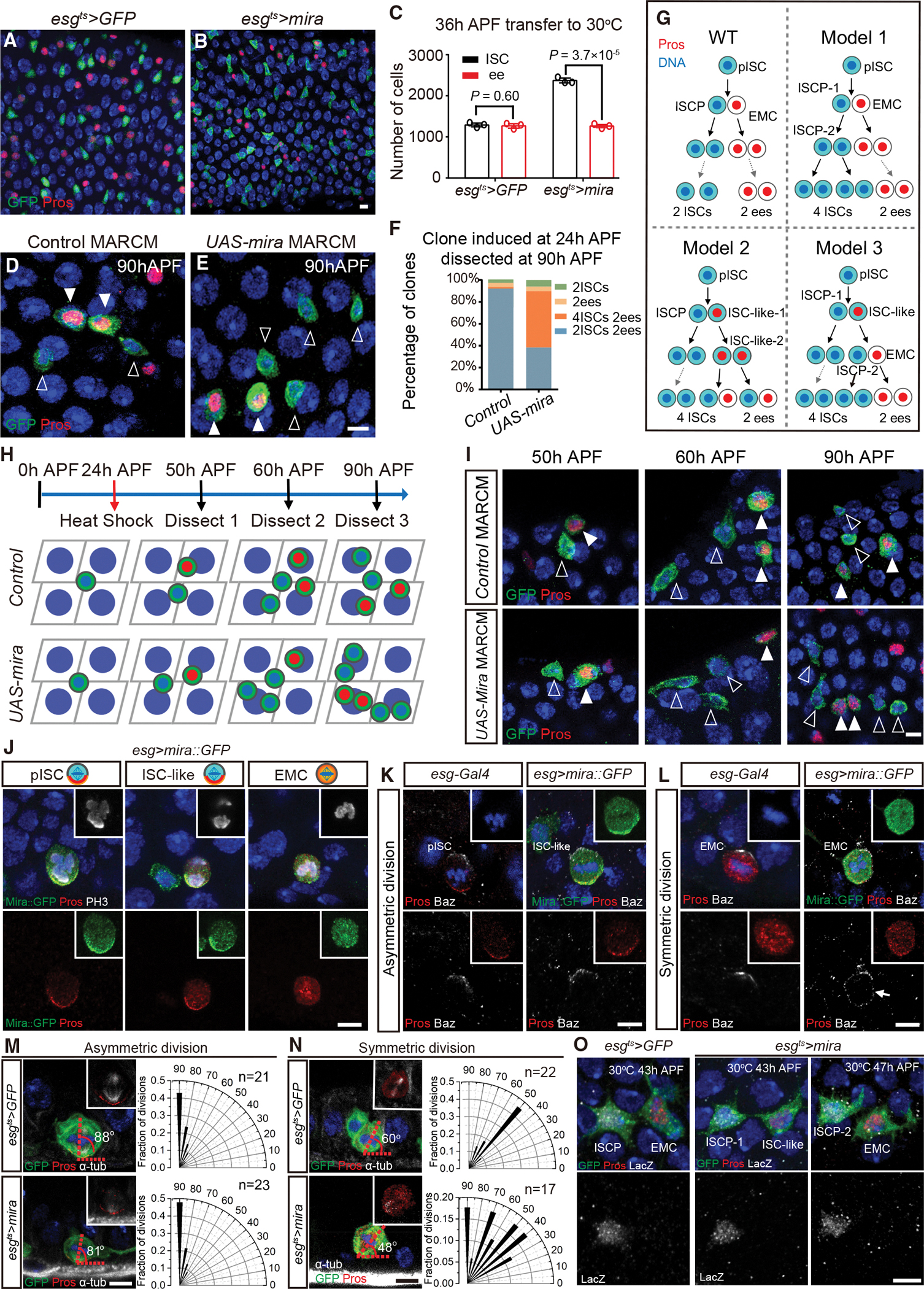
Ectopic Mira expression converts the pISC basal daughter to an ISC-like cell (A–C) *esg*^*ts*^>*mira* results in a significant increase in ISC (GFP^+^ Pros^−^) number, while the number of ee cells (Pros^+^) remains unchanged. n = 3 in each group. Data are presented as mean ± SD. (D and E) Representative control (D) and *UAS-mira* (E) MARCM clones, which were induced at 24 h APF and examined at 90 h APF. (F) Quantification of the frequency of clones in (D) and (E). Control, n = 76. *UAS-mira*, n = 99. (G) Three schematic potential models showing the way one pISC might produce four ISCs and two ee cells. (H and I) Schematic representation (H) and representative images (I) of control and *UAS-mira* MARCM clones induced at 24 h APF and examined at 50 h, 60 h, and 90 h APF. (J) In *esg>mira*::*GFP* intestines, Pros and Mira:GFP localize to the basal cortex and the cytoplasm of pISCs and ISC-like cells during mitosis. Pros and Mira:GFP is evenly distributed throughout the cytoplasm in dividing EMCs. (K) Baz localizes to the apical cortex when pISCs or ISC-like cells undergo asymmetric divisions in control (*esg-Gal4*) and *esg>mira*::*GFP* animals. (L) Baz forms an apical crescent in dividing control EMC. However, a uniform cortical Baz (arrow) is detected in dividing >*mira*::*GFP* EMC. (M and N) Representative images and radial histogram quantification of the division angles at metaphase of asymmetric (M) and symmetric (N) divisions in *esg*^*ts*^*>GFP* and *esg*^*ts*^>*mira* pupal midguts. n denotes scored cell number. (O) The Notch signaling reporter *NRE-lacZ* is present in ISCP in *esg*^*ts*^*>GFP* animals, and ISCP-1 and ISCP-2 in *esg*^*ts*^>*mira* animals. Scale bars, 5 μm.

To test this hypothesis, *esg*^*ts*^>*UAS-mira* flies (>*mira*) were examined. While the number of ISCs (GFP^+^ Pros^−^) was the same as the number of ee cells (Pros^+^) in *control* intestines (Figures 2A and 2C), ISC number was doubled and ee cell number was not changed in >*mira* midgut compared with the control intestine (Figures 2B and 2C). In addition, MARCM clones over-expressing Mira were induced at 24 h APF and examined at 90 h APF. At 90 h APF, 51% of *UAS-mira* MARCM clones contained six cells made up of four ISCs and two ee cells (Figures 2E and 2F), whereas 92% of control MARCM clones contained four cells consisting of two ISCs and two ee cells (Figures 2D and 2F). Our results suggest that ectopic Mira expression in pISC lineage results in more ISCs but not a tumor.

Why did Mira ectopic expression result in doubling of the ISC number? We developed three models that could account for an increase in ISC number without any changes in ee cell number (Figure 2G). To determine which model was correct, *control* and *UAS-mira* MARCM flies were heat-shocked at 24 h APF and dissected at 50 h, 60 h, and 90 h APF (Figure 2H). These results revealed that 90% of the *UAS-mira* clones were made up of 2-cell clones containing one Pros^−^ and one Pros^+^ cell at 50 h APF (Figures 2I and S3A). However, 25% of *UAS-mira* clones were 4-cell clones containing three Pros^−^ cells and one Pros^+^ cell at 60 h APF (Figures 2I and S3B). Since Model 3 fitted our results better (Figure 2G), our clone-tracing data suggested that >*mira* in the pISC lineage converts the pISC-derived EMC to an ISC-like cell (Figure S3C). This ISC-like cell asymmetrically divides, generating an additional ISCP (ISCP-2) and an EMC. ISCP-2 produces two additional ISCs, while EMC produces two ee cells (Figure S3C).

Our next doubt was why the EMC could not be converted into a second ISC-like cell in the >*mira* lineage. To answer this question, *esg>mira*::*GFP* flies were examined. Mira:GFP and Pros were co-localized to the basal cortex and the cytoplasm in pISCs and ISC-like cells under division (Figure 2J). However, Mira-Pros were uniformly distributed in the EMC cytoplasm (Figure 2J), suggesting that EMCs precluded the localization of Mira-Pros on the cell membrane. To test whether this was caused by a defect in cell polarity, Baz was stained in the *control* and >*mira*::*GFP* pupal intestines. Our results revealed that while Baz formed an apical crescent during the metaphase in >*mira*::*GFP* ISC-like cell as the *control* (Figure 2K), Baz was uniformly localized to the cell membrane of dividing EMCs in >*mira* midguts (Figure 2L). Correspondingly, while the division angles were normal in >*mira* pISCs (Figure 2M), >*mira* EMC division angles were randomly distributed (Figure 2N). Our results suggest that >*mira* EMC membrane are occupied by Baz, resulting in loss of cell polarity and, therefore, symmetric division in the presence of Mira.

On the other hand, Notch activation was observed in ISCP in *control* pupal midguts and ISCP-1/−2 in >*mira* pupal midguts (Figure 2O), and no difference in the level of activation was found (Figure S3D), indicating that ISCP identity is maintained by the Notch signaling. To further illustrate that Notch signaling is required for >*mira*-induced additional ISC maintenance, we co-expressed *Notch RNAi* and Mira in pISC lineages. Knockdown Notch forced all the >*mira* pISCs to differentiate into Pros^+^ cells (Figures S3E and S3F), suggesting that Notch is indeed activated in >*mira* apical daughters.

In conclusion, our results suggest that the >*mira* ISC doubling process results from the conversion of pISC basal daughters into ISC-like cells (Figure S3C).

### *mira* mutant pISC basal daughter is converted to an ISC-like cell

**Figure 3.**
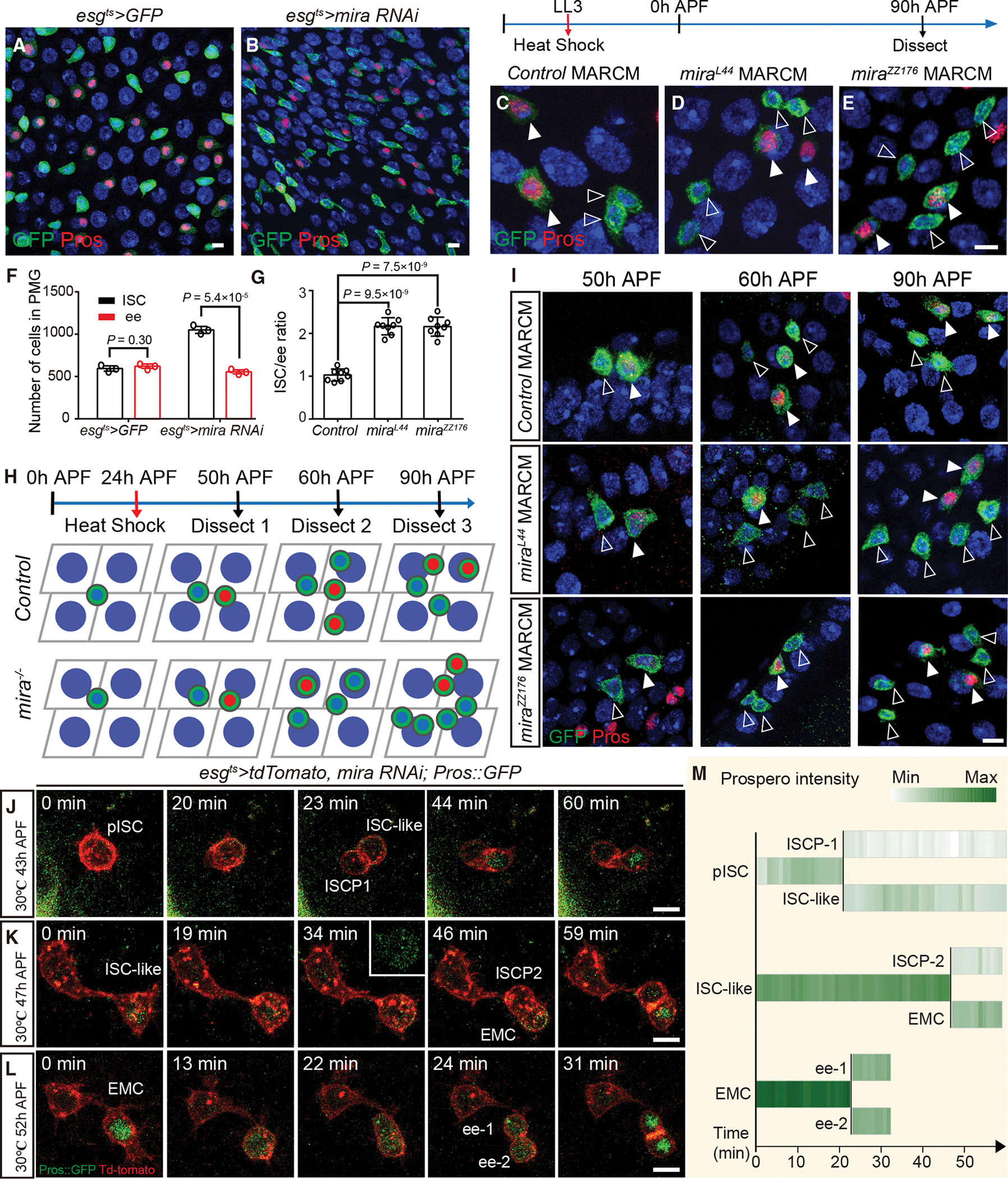
*mira* mutant pISC basal daughter is converted to ISC-like cell (A and B) ISC number is doubled and ee cell number is unchanged in *esg*^*ts*^>*mira RNAi* pupal midgut compared with *esg*^*ts*^*>GFP*. (C–E) Representative images of control (C), *mira*^*L44*^ (D), and *mira*^*ZZ176*^ (E) MARCM clones, which were induced at LL3 and examined at 90 h APF. (F) Quantification of the ISCs and ee cells in the *esg*^*ts*^*>GFP* and *esg*^*ts*^>*mira RNAi* posterior midguts (PMG). n = 3 in each genotype. (G) Statistics of ISC/ee ratio referred to in (C) to (E). The ratio was calculated by counting all clone cells in one gut. Eight guts with MARCM clones were counted for each genotype. (H and I) Schematic representation (H) and representative images (I) of *control* and *mira*^−/−^ MARCM clones induced at 24 h APF and examined at 50 h, 60 h, and 90 h APF. (J–L) Single frames of time-lapse movies of pISC (J), ISC-like cell (K) asymmetric division, and EMC symmetric division (L) after *mira* knockdown from LL3. See also Videos S4, S5, and S6. (M) Diagrams depicting changes in the intensity of Pros:GFP in ISC, ISC-like, EMC, and their progenies over time according to the frames in (J) to (L). Green represents Pros expression according to the scale (white, low; dark green, high). Data are presented as mean ± SD. Scale bars, 5μm.

Because the lack of *mira* expression in EMCs correlated with symmetric distribution of Pros (Figures 1E and 1F), we hypothesized that *mira*-deficient pISCs would be lost due to the symmetric distribution of Pros. However, after knockdown of *mira* expression in pISCs and their progeny (*esg*^*ts*^>*mira RNAi*), ISC number was almost doubled while ee cell number was unchanged in the posterior pupal midgut (Figures 3A, 3B, and 3F). To confirm the *mira RNAi* knockdown phenotype, *control*, *mira*^*L44*^, and *mira*^*ZZ176*^ null MARCM clones were induced at LL3 and examined at 90 h APF (Figures 3C–3E). In control animals, 18/24 4-cell clones contained two ISCs and two ee cells (Figure 3C). However, 21/24 *mira*^*L44*^ and 17/19 *mira*^*ZZ176*^ mutant clones consisted of 6-cell clones containing four ISCs and two ee cells (Figures 3D and 3E). In support of this observation, counting all of the cells in clones in the whole midgut revealed that while the ISC-to-ee ratio was approximately 1 in *control* clones, the ratio was approximately 2 in *mira*^*L44*^ and *mira*^*ZZ176*^ clones (Figure 3G). Therefore, contrary to our prediction, *mira*-deficient midguts have 1-fold more stem cells, reminiscent of the phenotypes observed with *mira* overexpression.

**Figure 4.**
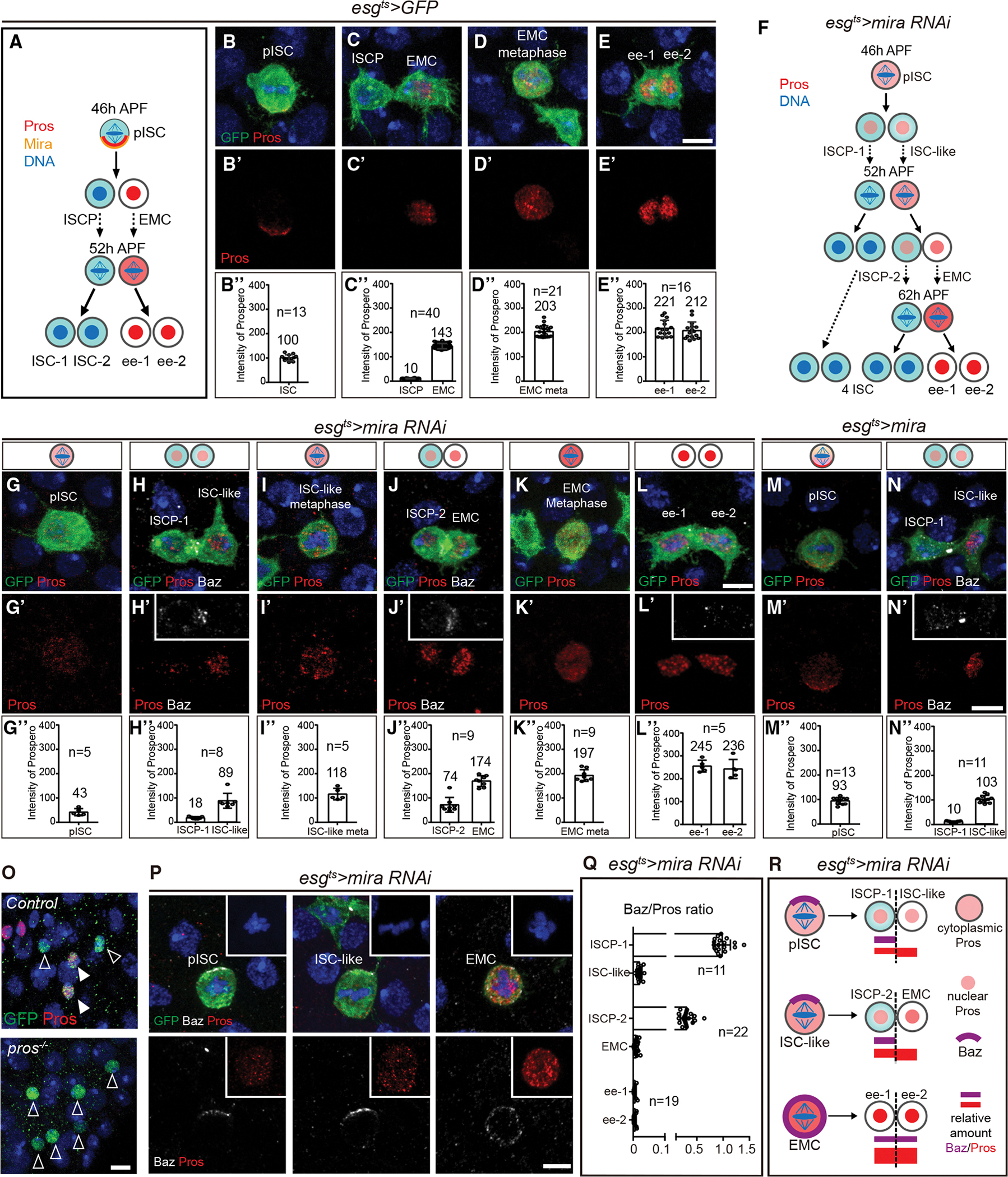
Lacking a threshold Pros level, pISC basal daughters become ISC-like cells (A) Schematic representation of the division pattern of pISC in *esg*^*ts*^*>GFP* pISC lineage. (B–E) Representative images and quantification of the intensity of Pros staining at metaphase in pISC (B), in a pair of ISCP and EMC after cytokinesis of pISC (C), in EMC at metaphase (D), and in a pair of ee cells after cytokinesis of EMC (E) in *esg*^*ts*^*>GFP* pISC lineage. The average intensity of Pros staining (AIPS) at metaphase of pISC is considered as 100. n denotes measured sample. (F) Schematic representation of the division pattern of the pISC lineage after *mira* knockdown. (G–N) Representative images and quantification of the intensity of Pros staining in pISC lineage after *mira* knockdown or *mira* overexpression. (O) Representative images of *control* and *pros*^17^ MARCM clones induced at 0 h APF and examined at 90 h APF. (P) Baz staining at metaphase in pISC lineage after *mira* knockdown. The cortical staining in the EMC is evident (n = 17). (Q) Baz intensity/Pros intensity ratio in daughter pairs after pISC, ISC-like, and EMC cell divisions in *mira* knockdown animals. The average Baz/Pros ratio at metaphase of ISCP-1 cells is considered as 1.0. n denotes scored pairs. (R) Model showing that Baz antagonizes Pros accumulation in the apical daughter (ISCP-1 and ISCP-2), and Pros has to achieve a threshold to establish the EMC identity. Dashed line indicates the cleavage furrow. Data are represented as mean ± SD. Scale bars, 5 μm.

To determine the ontogeny of the lineage of 6-cell mira mutant clones, *control*, *mira*^*L44*^, and *mira*^*ZZ176*^ MARCM clones were generated at 24 h APF and analyzed at 50 h, 60 h, and 90 h APF (Figure 3H). The benefit of clone induction at 24 h APF is to avoid MARCM clones labeling enterocytes,^52^ but the consequence is that Mira will have some perdurance in pISC lineage, therefore resulting in a weaker phenotype. At 50 h APF, most of the *control* and *mira* mutant clones contained two cells consisting of one Pros^+^ and one Pros^−^ cell (Figures 3I and S3G). At 60 h APF, approximately 10% of *mira* mutant clones contained four cells made up of three Pros^−^ and one Pros^+^ cell (Figures 3I and S3H). At 90 h APF, approximately 20% of *mira* mutant clones contained six cells containing four ISCs and two ee cells (Figures 3I and S3I). These results indicate that, as with >*mira*, *mira* mutants convert the pISC-derived EMC into an ISC-like cell, forming 6-cell pISC lineage containing four ISCs and two ee cells (Figure 4F).

How could binary daughter cell fates be generated in the absence of Mira? To answer this question, time-lapse movies of *mira* knockdown pupal midguts were taken at 43 h, 47 h, and 52 h APF at 30°C (Figures 3J–3L; Videos S4, S5, and S6), and the change in Pros:GFP intensity in the mother and the daughter cells was quantified over time (Figure 3M). In these movies, cytoplasmic Pros:GFP was evenly distributed before telophase in *mira*-deficient pISCs and ISC-like cells. However, Pros:GFP intensity became stronger in one daughter cell and weaker in the other after telophase, and nucleic Pros was unevenly distributed after cytokinesis (Figures 3J, 3K, and 3M; Videos S4 and S5). During *mira*-deficient EMC divisions, strong cytoplasmic Pros:GFP was symmetrically segregated between two daughters (Figures 3L and 3M; Video S6). Notably, the intensity of Pros:GFP was much higher in the EMC than that in ISC-like cell (Figure 3M), suggesting that the levels of Pros in the pISC basal daughter is critical for cell-fate determination. This result motivated us to quantify and compare the intensity of Pros in the pISC lineage under different genetic manipulations.

### Lacking a threshold Pros level, pISC basal daughters become ISC-like cells

To quantify and compare Pros expression, the average intensity of Pros staining (AIPS) in control pISCs at metaphase were set at a value of 100 (Figures 4A and 4B). AIPS was significantly increased to 143 in the EMC immediately after pISC asymmetric divisions. AIPS was further increased to 203 at metaphase of dividing EMCs, and reached a peak value of 221 in mature ee cells (Figures 4B–4E). Therefore, before entering mitosis, the content of Pros protein in EMCs is much higher than that in pISCs.

Next, AIPS was measured in the pISC lineage upon *mira* knockdown (Figures 4F–4L). Our results revealed that AIPS in ISC-like cells was significantly less than that in control EMCs (89 ± 31 versus 143 ± 11, p < 0.0001, Figures 4C and 4H). Thereafter, ISC-like-cell-derived EMCs achieved a higher level of Pros than that in *control* EMCs (174 versus 143, Figures 4C and 4J) and underwent symmetric divisions in the *mira* RNAi pISC lineage (Figures 4K and 4L). Taken together, these results suggest that a threshold of Pros (here as AIPS at an approximate value of 143) is required for pISC basal daughter differentiation into an EMC.

If the conversion of the pISC basal daughter to an ISC-like cell during *mira* knockdown is due to insufficient Pros allocation, we wondered whether this also occurred during *mira* overexpression. During >*mira* pISC metaphase, Pros was not only localized to the basal cortex but also was diffusely present in the cytoplasm (Figure 4M). As a result, the AIPS in the ISC-like cell was 103 (Figure 4N), lower than the threshold value of 143. Thus, Mira ectopic expression caused an insufficient Pros allocation in the pISC basal daughter, and this basal daughter was converted to an ISC-like cell. Moreover, the AIPS in the basal daughter of >*mira* ISC-like cell was 166 (>143), and as a result differentiated into an EMC (Figures S4A–S4D), further suggesting that a critical threshold of Pros is necessary for EMC differentiation.

To genetically demonstrate that the Pros level was critical for pISC basal daughter differentiation, *control* and *pros^17^* MARCM clones were induced at 0 h APF and examined at 90 h APF. While the majority of *control* clones were 4-cell clones containing two ISCs and two ee cells, 37% of *pros^17^* clones contained six ISCs (Figures 4O and S4E), indicating that *pros*-deficient pISC basal daughters underwent two rounds of cell divisions as opposed to one round of cell division in *wild-type* EMC.

We noted that Pros was segregated into ISCP-1 and ISCP-2 cells in *mira* knockdown pupal midgut (Figures 4H and 4J). Why did these cells not differentiate into Pros^+^ EMCs? It is worth noting that Baz formed an apical crescent at metaphase in *mira* knockdown pISCs and ISC-like cells (Figure 4P). By contrast, Baz was uniformly located on the cortex of mitotic EMCs (Figure 4P). Accordingly, division angles showed stochastic allocations in EMCs during metaphase (Figure S4F). More importantly, after cytokinesis, Baz was segregated into ISCP-1 and ISCP-2 (Figures 4H and 4J). By quantifying the Baz and Pros relative amount in *mira* knockdown pISC lineage, we found that a high Baz/Pros ratio correlated with stem cell fate, while a low Baz/Pros ratio correlated with ee cell fate (Figure 4Q). Therefore, we propose a model in which the Baz/Pros ratio determines sibling cell fates: when Pros levels fall below a threshold, Baz in apical daughters maintains stem cell fate by counteracting Pros accumulation during the first two asymmetric divisions. When EMCs undergo symmetric divisions, a high level of Pros promotes ee cell differentiation even in the presence of Baz (Figure 4R).

How does Baz counteract the Pros accumulation in ISCPs? In line with the Baz localization after mitotic divisions, we found that Notch activity was activated in the Baz^+^ ISCP-1 and ISCP-2 cells in pupal midgut after *mira* knockdown (Figures S4G and S4H). Furthermore, knockdown *Notch* turned all the *mira* knockdown pISC daughters into Pros^+^ ee cells (Figures S4I and S4J), suggesting that Notch was activated in *mira* knockdown pISC lineages. Therefore, Baz may facilitate the Notch activation in the ISCPs to inhibit Pros accumulation.

Taken together, our results suggest that, below a threshold of Pros level, pISC basal daughters become ISC-like cells. Under *mira* knockdown conditions, although Pros presents in the apical daughters, Baz may promote the stem cell fate specification through Notch signaling.

### Baz/Pros ratio determines daughter cell fate in *par-6* or *aPKC* mutant pISC lineage

**Figure 5.**
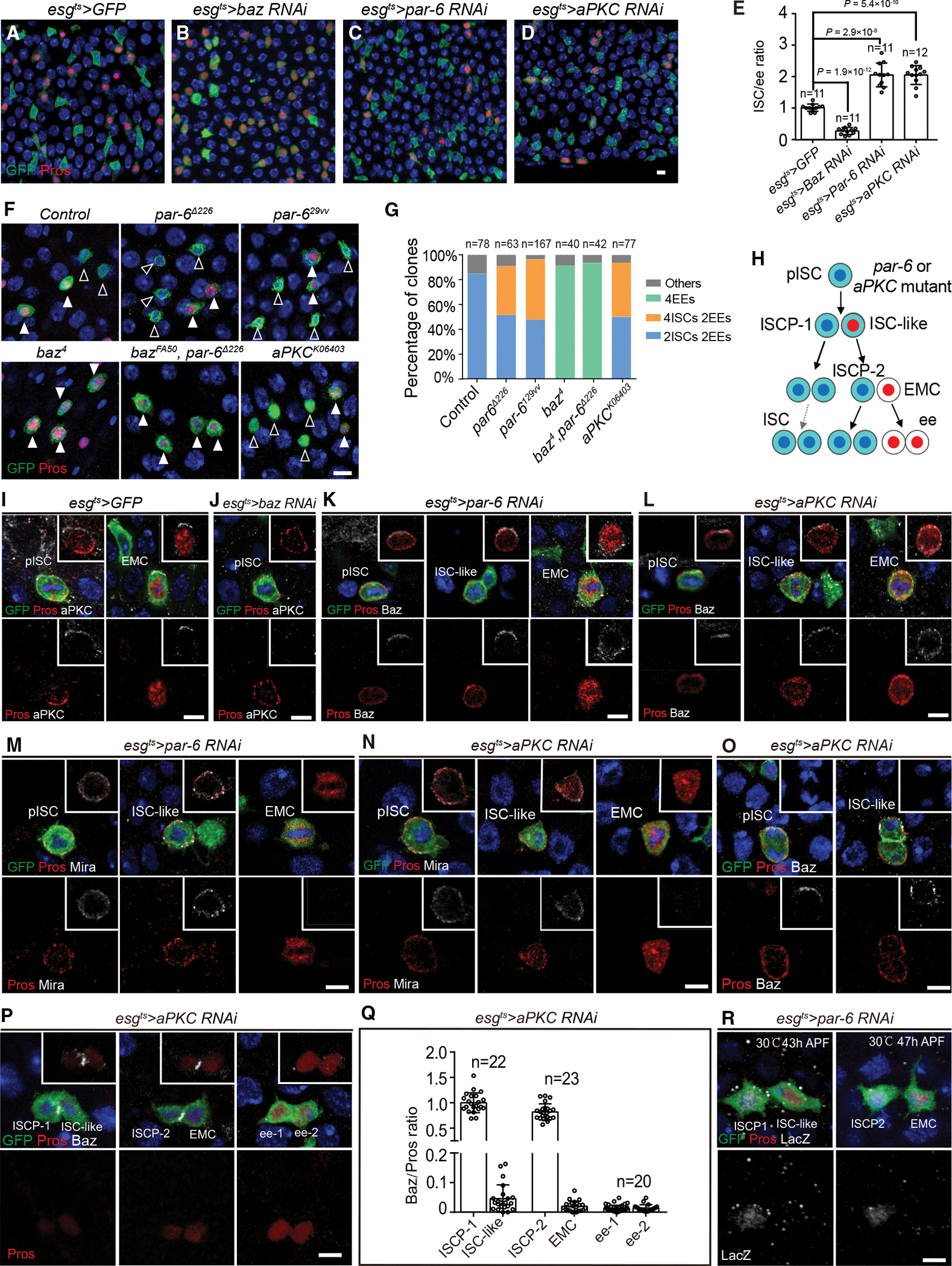
Baz/Pros ratio determines daughter cell fate in *par-6* or *aPKC* mutant pISC lineage (A–D) In contrast with control pupal intestines (A), the majority of pISC lineage with *baz* knockdown differentiate into ee cells (B), while knockdown of *par-6* (C) or *aPKC* (D) in pISCs doubles the ISC number. (E) Statistics of ISC/ee ratio of (A) to (D). n denotes scored gut number. (F) Representative *par-6*^*Δ226*^, *par-6*^*29vv*^, *baz*^*4*^, *baz*^*FA50*^ *par-6*^*Δ226*^, and *aPKC*^*K06403*^ MARCM clones induced at 0 h APF and examined at 90 h APF. (G) Statistics of the percentage of different clone types in (F). n denotes scored clones. (H) Schematic model showing the production of four ISCs and two ee cells from one pISC in *par-6* or *aPKC* mutant pISC lineage. (I) aPKC apical crescent at metaphase in pISC asymmetric division and EMC symmetric division in *esg*^*ts*^*>GFP* midguts. (J) Crescent aPKC is lost in *baz* knockdown pISC. (K and L) Baz forming apical crescent in *par-6* (K) *and aPKC* (L) knockdown dividing pISC and ISC-like cell, and Baz cortical localization in dividing EMC. (M and N) Mira and Pros evenly distributed on the cortex at metaphase of *par-6* (M) and *aPKC* (N) knockdown pISC and ISC-like cell. Mira expression disappears on EMCs in metaphase. (O) Pros is ubiquitously localized on the cell membrane either on ISC at telophase or ISC-like cell at cytokinesis in *esg*^*ts*^*>aPKC RNAi* pupal midgut. (P) Baz and Pros staining after pISC, ISCP, and EMC cytokinesis in *esg*^*ts*^*>aPKC RNAi* pupal midgut. (Q) Baz intensity/Pros intensity ratio in *aPKC RNAi* pISC lineages. The average Baz/Pros ratio in ISCP-1 is considered as 1.0. n denotes scored pairs. (R) *NRE-lacZ* is present in ISCP-1 and ISCP-2 in *esg*^*ts*^*>par-6 RNAi* animals. Data are presented as mean ± SD. Scale bars, 5 μm.

Since loss of Baz results in a significant ISC loss (Figures 5B and 5E), we hypothesized that the knockdown of the Par complex component Par-6/aPKC would also cause stem cell loss and an increase in ee cells. Unexpectedly, *par-6* or *aPKC* knockdown resulted in a significant increase in ISCs compared with *controls* (Figures 5A and 5C–5E). To confirm these RNAi knockdown results, *control*, *par-6*^*Δ226*^, *par-6*^*29vv*^, *baz*^*4*^, *baz*^*FA50*^ *par-6* ^*Δ 226*^, and *aPKC*^*k06403*^ MARCM clones were induced at 0 h APF and dissected at 90 h APF (Figure 5F). Over 80% of *control* clones were made up of four cells comprising two ISCs and two ee cells (Figures 5F and 5G). By contrast, 40%–50% of *par-6* or *aPKC mutant* MARCM clones were made up of six cells including four ISCs and two ee cells (Figures 5F and 5G), a phenotype seen with *mira* knockdown (Figures 3D, 3E, and 3G). However, most of the *baz* single mutant and *baz*, *par-6* double mutant clones consisted of four ee cells (Figures 5F and 5G), indicating that *baz* is epistatic to *par-6*. Together, our results demonstrate that while ISCs are lost in *baz* mutant conditions, ISC number is doubled in *par-6* or *aPKC* mutant midguts, suggesting that *baz* has other functions besides polarity.

To determine the reason why ISC number doubled in *par-6* or *aPKC* knockdown intestines, *par-6 RNAi* or *aPKC RNAi* MARCM clones were induced at 0 h APF and examined at 50 h, 60 h, and 90 h APF (Figures S5A–S5E). “3 Pros^−^ 1 Pros^+”^ MARCM clones were present in *par-6 RNAi* or *aPKC RNAi* pupal midguts at 60 h APF (Figures S5A, S5B, and S5D), suggesting that the knockdown of *par-6* or *aPKC* converts pISC basal daughters to ISC-like cells (Figure 5H), which generates additional ISCs.

We next asked why ISCs were lost in *baz* knockdown pISC lineages but doubled in *par-6* or *aPKC* knockdown pISC lineages. We therefore checked aPKC and Baz localization under different genetic backgrounds. First, in control midguts, aPKC localized to an apical crescent on dividing pISCs and EMCs (Figures 5I and S5F). Consistently, in >*mira* or *mira RNAi* pupal midguts, aPKC localized to the apical cortex of dividing ISCs and ISC-like cells and distributed on the cell cortex of dividing EMCs (Figures S5F–S5H), showing the same pattern as Baz localization (Figures 2K, 2I, and 4P). However, aPKC staining was lost in dividing pISCs after *baz* knockdown (Figure 5J, n = 31), indicating that aPKC localization depends on the localization of Baz. Next, we examined Baz localization in a *par-6* or *aPKC* knockdown background. Notably, Baz localized to an apical crescent in dividing pISCs and ISC-like cells and localized to the cell cortex of dividing EMCs in *par-6* (Figure 5K, n = 12, 16, 9) or *aPKC* knockdown pupal midguts (Figure 5L, n = 14, 15, 7), suggesting that Baz localization does not depend on the function of Par-6 or aPKC. Moreover, although Mira staining was not detected in dividing EMCs (Figures 5M and 5N), Mira co-localized with Pros on the cell cortex of dividing pISC and ISC-like cells in *par-6* (n = 27, 16) or *aPKC* (n = 30, 12) knockdown pupal midguts (Figures 5M and 5N), demonstrating that *par-6*/*aPKC* is required for Mira-Pros basal crescent formation. In summary, these results suggest that the apical exclusion of Mira-Pros depends on the function of Par-6 and aPKC. aPKC is recruited by the apical localization of Baz, but Baz localization does not depend on Par-6 or aPKC. Therefore, apical Baz localization might maintain ISC fate, and *par-6* or *aPKC* defects induce even Mira-Pros distribution, which in turn causes the increase in ISC number.

We next asked how evenly distributed Mira-Pros could generate binary cell fates and double the ISC number in *par-6* or *aPKC* knockdown pupal midguts. In a previous report on the neuronal system, *baz*/*aPKC* mutant NBs corrected earlier Mira-Pros localization defects during telophase.^10^ However, Mira-Pros was ubiquitously distributed on the cell membrane of *par-6* or *aPKC* knockdown pISCs and ISC-like cells at telophase and cytokinesis (Figures 5O and S5I), suggesting that binary cell fate is not caused by “telophase rescue.” To determine how binary cell fate was generated, time-lapse movies of *aPKC RNAi* pupal midgut were recorded and Pros:GFP intensity was quantified (Figures S5J–S5M; Videos S7, S8, and S9). Pros:GFP was evenly distributed on the cell membrane of pISC and ISC-like cell before cytokinesis (Figures S5J and S5K) and then symmetrically segregated into the nuclei of sibling cells during cytokinesis (Figures S5J and S5K). However, Pros:GFP disappeared in ISCPs at 1 h after mitosis (Figures S5J and S5M), suggesting that Pros expression cannot be maintained in ISCPs. Furthermore, to demonstrate that the ISC-like cell formation in *par-6* or *aPKC* knockdown lineage was caused by insufficient Pros allocation, AIPS was quantified in *aPKC RNAi* pISC lineages (Figures S5N–S5T), revealing that it was significantly lower in ISC-like cells than in EMCs (53 versus 141, p < 0.0001, Figures S5P and S5R). Taken together, these results suggest that, owing to insufficient Pros allocation into the basal side, pISC basal daughters that underwent *par-6*/*aPKC* knockdown are converted to ISC-like cells. On the other hand, Pros accumulation is inhibited in apical ISCPs, thus maintaining the stem cell identity.

Lastly, we asked how the apical daughter of pISCs antagonized the Pros accumulation. Given that Baz localization was not affected by the functions of *par-6*/*aPKC*, pICS lineage underwent *par-6*/*aPKC* knockdown provides us with a scenario to further test the hypothesis that the Baz/Pros ratio specifies the fate of siblings. We observed that Baz localized on ISCP-1 and ISCP-2 cells, and weakly on both ee cells, after mitotic divisions in *aPKC RNAi* pISC lineages (Figure 5P). The quantification of Baz/Pros showed that a high ratio correlated with antagonization of Pros accumulation thereby establishing the ISCP cell fate, and a low ratio correlated with ee cell fate (Figure 5Q). Consistent with the idea that Baz promotes Notch signaling, Notch signaling was activated in the Baz^+^ ISCP-1 (n = 20) and ISCP-2 (n = 20) cells in pupal midguts with *par-6* knockdown (Figure 5R). In summary, our results support the idea that the Baz/Pros ratio determines daughter cell fate and hint at the possibility that Baz facilitates Notch activation to block Pros accumulation after cytokinesis.

### Baz facilitates Notch activation through recruiting Notch receptor to the interface

**Figure 6.**
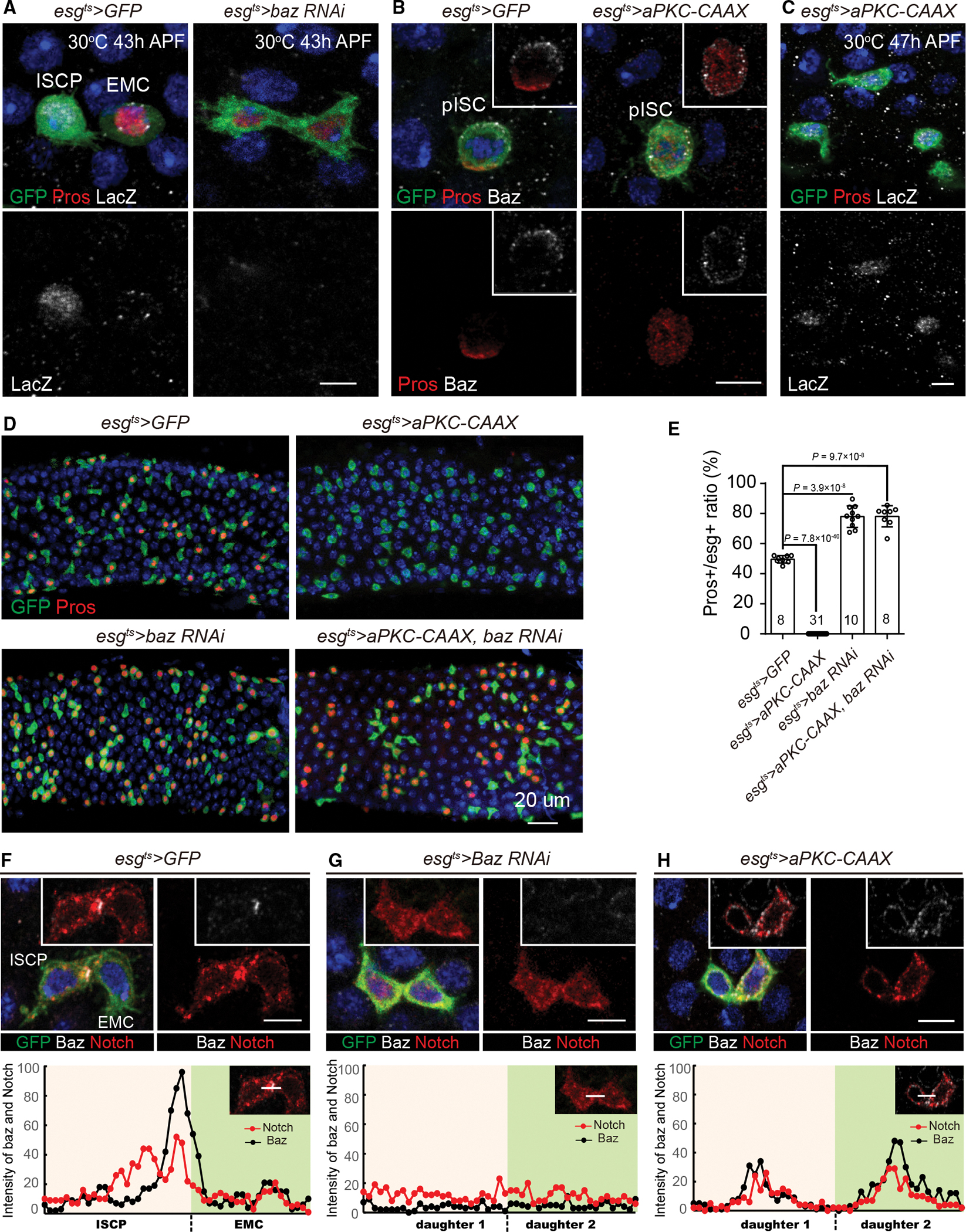
Baz promotes Notch activation through recruiting Notch receptor to the interface (A) *NRE-lacZ* is present in ISCP in *esg*^*ts*^*>GFP* animals, while *NRE-lacZ* staining is absent in *esg*^*ts*^*>baz RNAi* pISC lineage. (B) Baz forming apical crescent in *esg*^*ts*^*>GFP* dividing pISC, and Baz ubiquitous localization on the cell membrane of dividing *esg*^*ts*^*>aPKC-CAAX* pISC. (C) *NRE-lacZ* staining is present in each *esg*^*ts*^*>aPKC-CAAX* pISC lineage cell. (D) Compared with *esg*^*ts*^*>GFP*, *esg*^*ts*^*>aPKC-CAAX* pupal midgut has no Pros^+^ ee cells at 90 h APF, while most of the esg^+^ cells in *esg*^*ts*^*>baz RNAi* pupal midguts are Pros^+^ ee cells. Knockdown *baz* in *esg*^*ts*^*>aPKC-CAAX* pupal midguts shows *baz* mutant phenotype. Scale bar, 20 μm. (E) Statistics of Pros^+^:*esg*^+^ ratio referred to in (D). The ratio was calculated by counting cells in a 20× image of posterior pupal midgut. Number of guts counted is indicated in the column. (F–H) Baz and Notch staining in post-dividing daughters in *esg*^*ts*^*>GFP* (F), *esg*^*ts*^*>baz RNAi* (G), and *esg*^*ts*^*>aPKC-CAAX* (H) pupal midgut. Baz and Notch fluorescence distributions crossing the interface (white bar in the upper-right corner of graphs) were measured. Data are presented as mean ± SD. Scale bars, 5 μm.

To find direct evidence that Baz facilitates Notch activation, we examined the Notch activity in *baz* knockdown pupal midguts. Our results showed that the Notch signaling reporter *NRE*-LacZ was absent in *baz* knockdown pISC daughters (Figure 6A, n = 120), indicating that Baz is necessary for Notch activation. To demonstrate that the differentiation of ee cells caused by *baz* knockdown was caused by the lack of Notch activation, we ectopically expressed a constitutive active form of Notch receptor, Notch^ECN^,^53^ under the *baz* knockdown background (Figure S6A). Notch activation in *baz* knockdown pISC lineage fully inhibited the differentiation of pISC progenies into ee cells (Figures S6A and S6B), demonstrating that the *baz* knockdown phenotype is caused by Notch inactivation.

To corroborate that Baz is sufficient for Notch activation, we ectopically expressed the constitutively active aPKC construct *UAS-aPKC-CAAX* in pISC lineage (>*aPKC-CAAX*)^54^ and found that Baz was localized on the cortical, while Pros localized in the cytoplasm, in >*aPKC-CAAX* dividing pISCs (Figure 6B, n = 30). Consistent with the idea that Baz facilitates Notch activation, we observed that *NRE*-LacZ was in >*aPKC-CAAX* cells (Figure 6C, n = 100). As a consequence, no Pros^+^ ee cells were observed in >*aPKC-CAAX* midguts (Figures 6D and 6E). To prove that >*aPKC-CAAX* indeed induced Notch activation, we knocked down Notch in >*aPKC-CAAX* midguts. *Notch RNAi*, >*aPKC-CAAX* progenies showed the *Notch RNAi* phenotype (Figures S6A and S6B), suggesting that the phenotype caused by >*aPKC-CAAX* was caused by Notch activation. To demonstrate that the activation of the Notch signaling caused by >*aPKC-CAAX* was caused by the recruitment of Baz to the cortical membrane, we ectopically expressed aPKC-CAAX while additionally knocking down Baz, and almost all pISCs differentiated into Pros^+^ ee cells (Figures 6D and 6E). Together, our results suggest that Baz is necessary and sufficient for Notch activation in pISC lineages.

What might be the molecular mechanism by which cortical Baz facilitates Notch activation? We noticed that after the asymmetric division of pICS/ISCPs, Baz located to the apical daughter at the interface where two siblings touch (Figures 4H, 4J, 4N, and S4B). Since activation of Notch signaling is due to proteolytic cleavages of Notch receptors at the cell membrane caused by ligands delivered by adjacent cells that change their conformation,^55–58^ we speculated whether it was Baz that enriched Notch receptors to the interface. Consistent with our speculation, Notch receptor staining coincided with the Baz localization after the pISC asymmetric divisions (Figures 6F and S6C, n = 22). In addition, knockdown of Baz abolished the Notch receptor aggregation at the interface and increased the distribution of Notch receptor in the cytoplasm (Figures 6G and S6D, n = 28). Moreover, >*aPKC-CAAX* resulted in a uniform distribution of Notch receptors across the cell membrane of both daughters, with co-localization upon Baz staining (Figures 6H and S6E, n = 31). Collectively, Baz may facilitate Notch activation by recruiting Notch receptors to the interface where the two daughters come into contact.

### G_1_ Pros expression precedes G_2_ Mira expression, and Pros inhibits the expression of Mira after reaching the threshold

Our last question is, why is Mira expressed in pISC along with Pros, whereas Mira expression is suppressed in EMC? Since Pros binds to the promoter region of *mira* and represses *mira* transcription in NBs,^59^ we hypothesized that Mira is expressed earlier in the mitotic cell cycle of pISCs than Pros, bringing Pros to the basal membrane to avoid transcriptional inhibition. However, in *par-6/aPKC* knockdown ISC-like cells, while Pros was inherited from pISC asymmetric divisions, Mira was still expressed (Figures 5M and 5N), suggesting that further investigation is required.

**Figure 7.**
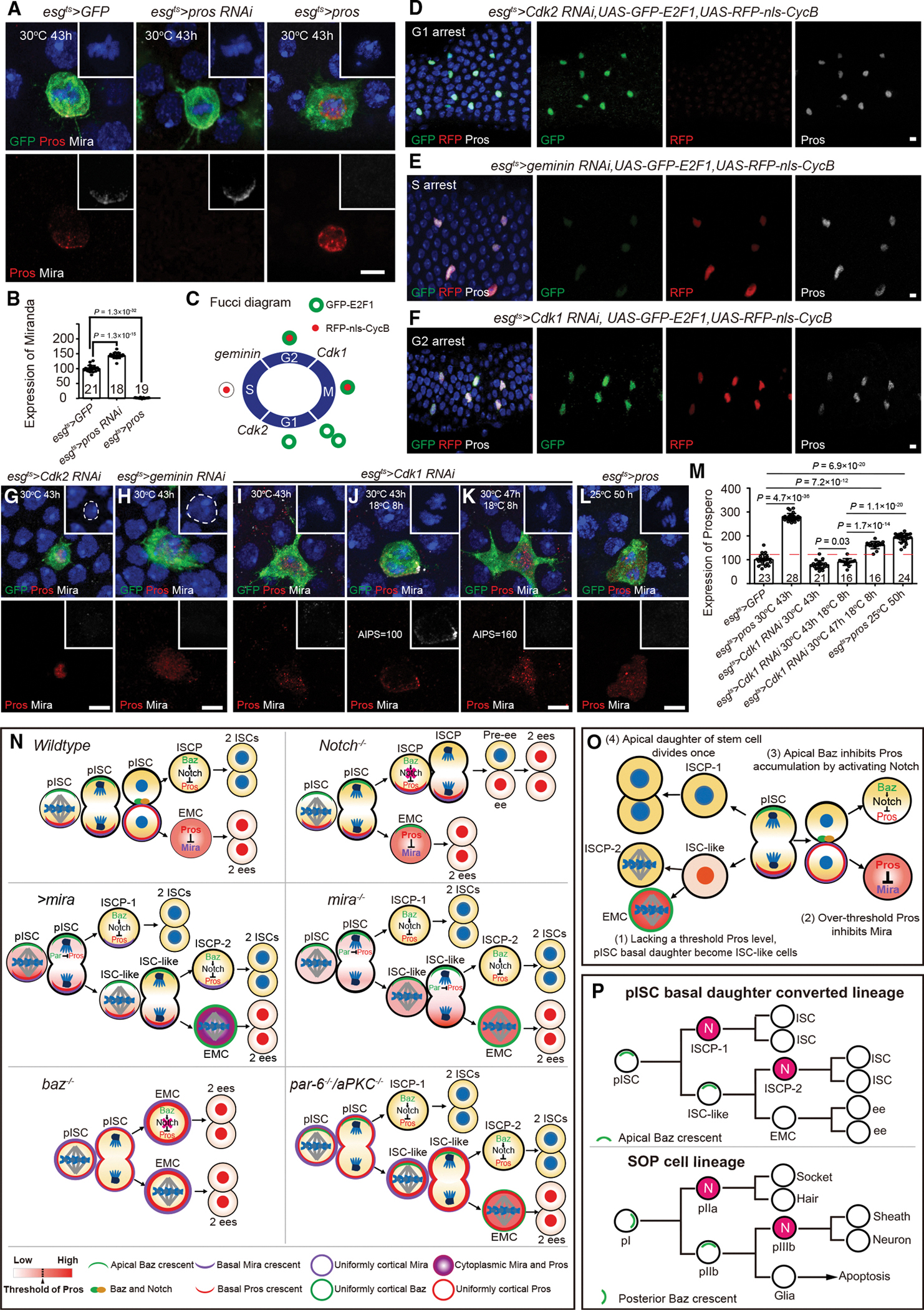
G1 Pros expression precedes G2 Mira expression, and Pros inhibits the transcription of Mira after reaching the threshold (A) Mira is upregulated in *pros* knockdown dividing pISC and is absent in *>pros* dividing pISC, in contrast with Mira in the *control* dividing pISC. (B) Quantification of the intensity of Mira staining in (A). The number of measured samples is indicated in the columns. (C) Diagram of the Fucci system used in the pISC lineage. In G_1_ phase, only GFP-E2F1 is present in the cytoplasm. As cells progress from G_1_ to S phase, GFP-E2F1 is degraded and nuclear RFP-nls-CycB is present. From G_2_ to M phase, both GFP-E2F1 and RFP-nls-CycB are accumulated in the pISCs. (D–F) Revealed by Fucci system, knockdown *Cdk2*, *geminin*, and *cdk1* in pISCs induces G_1_ (D), S (E), and S_2_ (F) phase arrest. All the cell-cycle-arrested pISCs are Pros^+^. (G–I) Pros and Mira staining in pISCs subjected to *Cdk2* RNAi (G), *geminin* RNAi (H), or *Cdk1* RNAi (I) at 30°C 43 h APF. Cell-cycle-arrest pISCs are accumulating Pros with no Mira expression. (J and K) Pros and Mira staining in *Cdk1* RNAi flies, which were kept at 30°C for 43 h (J) or 47 h (K) before being transferred to 18°C for 8 h. (L) Pros and Mira staining in *esg*^*ts*^*>pros* dividing pISC at 25°C 50 h APF. (M) Quantification of Pros expression at the indicated conditions. The red dashed line indicates the threshold of Pros-suppressing Mira expression when Pros intensity was above this line. (N) Detailed schematic representation of pISC lineages under different genetic manipulations (see also the main text). (O) Four principles illustrating how ISC number is precisely determined in a pISC lineage. (P) Similarities between EMC converted pISC lineage and sensory organ precursor (SOP) lineage. N represents Notch activation. Green crescent indicates the Baz localization, and represents two types of asymmetric divisions (pI, anterior-posterior; pIIb, apical-basal) in SOP cell lineage. Data are presented as mean ± SD. Scale bars, 5 μm.

Consistent with the theory that Pros transcriptionally represses *mira* expression, Mira staining was markedly increased in dividing pISCs with *pros* knockdown (Figures 7A, 7B, S7A, and S7B). By contrast, overexpression of Pros completely inhibited Mira expression in pISCs at 43 h APF at 30°C (Figures 7A and 7B). Therefore, Pros downregulates *mira* expression in pISCs.

To identify the expression order of Pros and Mira, we used RNAi to block the cell cycle in each specific phase. Through the Fly-FUCCI system (Figure 7C),^60^ our results showed that knockdown of *Cdk2*,^61^ *geminin*,^62^ and *Cdk1*^63^ resulted in pISC staying in the G_1_, S, and G_2_ phases of the cell cycle, respectively (Figures 7D–7F). Notably, Pros staining was positive in all cell-cycle-arrested pISCs (Figures 7D–7F and S7C–S7G), implying that Pros expression starts as early as the G_1_ phase of pISC division and accumulates over the cell cycle.

Next, we examined the expression of Mira in pISCs stalled in G_1_, S, and G_2_ phases, respectively. We found no Mira staining in any of the cell-cycle-stalled pISCs at 43 h APF, when asymmetric division is supposed to begin (Figures 7G–7I and S7H), suggesting that Mira expression is later than G_2_ phase, which is stalled by knockdown of *Cdk1* (Figure 7I). Interestingly, when we took the pupae that knocked down *Cdk1* at 30°C for 43 h back to 18°C for 8 h, thus allowing *Cdk1* to be expressed in the pISCs, driving the cells into M phase, at which point we observed Mira and Pros localized to the basal crescent in the dividing pISCs (Figures 7J and S7H). However, when we placed the *Cdk1* knockdown pupae at 30°C for 47 h and then back to 18°C for 8 h, no Mira protein levels could be observed in the pISCs at this time (Figures 7K and S7H), indicating that the persistent accumulation of Pros exceeds a threshold that suppressed Mira expression. To further prove our speculation and to rule out that Mira expression was not observed due to not entering the M phase, we used *esg*^*ts*^ to mildly overexpress Pros at 25°C to 50 h APF, when pISC entered mitosis, whereby we found that the excess Pros completely suppressed Mira staining (Figures 7L and S7H), thus indicating that a threshold-exceeding Pros expression level suppresses *mira* expression.

Our data suggest that G_1_ Pros expression precedes G_2_ Mira expression, and if we quantified the expression level of Pros we found that when the expression of Pros exceeded a threshold in pISC it repressed the expression of *mira* (red dashed line in Figure 7M). In summary, we propose a dynamic interlocked model in which Pros expression in pISCs initiates during the G_1_ phase, followed by Mira expression during the late G_2_ phase. Mira then targets Pros to the basal membrane to avoid transcriptional inhibition. Following asymmetric division of pISCs, the over-threshold Pros inhibits *mira* transcription in the EMC, resulting in symmetric segregation of Pros and the generation of two ee cells (Figure S7I).

## DISCUSSION

How the number of epithelial stem cells is determined during tissue development remains unclear. Our work revealed that the pISC first asymmetrically divides, generating one apical Baz^+^ ISCP and one basal Pros^+^ EMC. Apical Baz facilitates Notch activation by recruiting Notch receptor to the interface where two siblings touch, which represses the transcription of Pros in the ISCP, resulting in two ISCs. Over-threshold of Pros in the EMC inhibits *mira* expression, resulting in the symmetric division of EMC and the formation of two ee cells (Figure 7N, *wild-type*). The apical-basal polarity in *Notch* mutant pISC is not affected. As a result, ISCP-EMC siblings are still generated following asymmetric division of *Notch* mutant pISCs. However, ISCP cannot inhibit Pros expression in the absence of Notch and undergoes an extra round of asymmetric division, leading to the production of a pre-ee and an ee. Pros accumulates in the pre-ee and eventually becomes an ee cell (Figure 7N, *Notch*^−/−^). >*mira* and *mira* mutants result in the same ISC doubling phenotype (Figure 7N, >*mira* and *mira*^−/−^). Overexpression of Mira or *mira* mutants dilutes the concentration of Pros in the basal progeny, thereby converting pISC basal daughters into ISC-like cells due to not reaching the threshold level of Pros. By contrast, Pros accumulates and achieves the threshold in the basal daughter of the ISC-like cell, and this daughter loses polarity and becomes an EMC. Therefore, EMC symmetrically divides even in the presence of ectopic Mira (Figure 7N, >*mira*). On the other hand, since Baz is required for Notch activation, the *baz*^−/−^ pISC lineage give rise to four ee cells with the same phenotype as *Notch*^−/−^ (Figure 7N, *baz*^−/−^). It is worth noting that Baz apical localization is not affected in *par-6/aPKC* mutant pISC lineage, and Baz ensures the fate of ISCP cells by activating Notch. However, *par-6/aPKC* mutation causes the Mira-Pros complex to be located on the cortex, decreasing the concentration of Pros in the basal progeny, allowing the pISC basal progeny to be transformed into an ISC-like cell, and eventually doubling the number of ISCs (Figure 7N, *par-6*^−/−^ or *aPKC*^−/−^).

Based on these six types of pISC lineages, four principles are proposed to illustrate how ISC number is precisely determined (Figure 7O): (1) lacking a threshold Pros level, pISC basal daughters become ISC-like cells; (2) over-threshold Pros inhibits *mira* transcription, thus leading to symmetric division; (3) apical Baz inhibits Pros accumulation by activating Notch; and (4) apical daughters of pISCs or ISC-like cells only divide once (thus, pre-ee in *Notch*^−/−^ cells do not divide). These principles collectively determine the pattern of pISC lineage and the final number of ISCs.

Both NBs and pISCs use Baz/Par-6/aPKC to define the apical-basal polarity and to distribute Mira and Pros on the basal cell membrane during mitotic division.^64^ After asymmetric division, both GMCs and EMCs divide once more. Notably, our finding that the ratio of Baz to Pros determines the fate of pISC progeny is also shown to hold true in the NB lineage, where an excess amount of Pros allows progeny that acquired Baz to differentiate into GMCs.^65^ However, asymmetric divisions of pISCs are different from that of NBs in a number of ways: (1) the apical daughters of pISCs return to the basal membrane to maintain a single layer of epithelium in the pupal intestine, whereas the NB remains in an apical position following mitosis;^11,12,66^ (2) siblings generated by asymmetric division of pISCs are equal in cell size, whereas NBs give rise to a larger NB and a smaller GMC;^67,68^ and (3) the apical daughters of pISCs symmetrically divide once more, generating two ISCs, whereas NBs continue undergoing asymmetric divisions.^7,69^

So how are we to understand the differences between pISC and NB lineages? In the present study, the pISC basal daughter converted lineage underwent three rounds of mitosis and showed a pattern comparable with that of the SOP lineage (Figure 7P): (1) similar to the pISC lineage, which forms a monolayer epidermis, the asymmetric division of pI in the SOP lineage forms an anterior-posterior polarity, leaving pIIa and pIIb in the same plane;^70^ (2) siblings of pISCs and pI do not have a significant size difference after asymmetric division;^67,70^ and (3) the first two rounds of asymmetric division in both lineages result in Notch activation in the Baz^+^ progeny,^47,71,72^ whereas two Notch-signaling-positive cells in the pISC lineage form four adult ISCs while two Notch signaling active cells in the SOP lineage form an external sensory organ.^70^ Thus, the differences between the pISC and NB lineages are precisely the similarities between the pISC and SOP lineages, and the pISC lineage can be considered as an intermediate state between the NB and SOP. Future studies of the molecular mechanism of pISC asymmetric division will build a bridge to a deeper understanding of NBs and SOPs.

This study found that Baz, but not Numb, plays a key role in the uneven Notch activation of the pISC lineage. Although we observed an asymmetric distribution of Numb in dividing pISCs (Figure S2B), neither knockdown of Numb nor two different *numb* mutants caused Notch activation in EMCs (Figures S2D and S2F). We speculate that the asymmetric activation of Notch in pISC daughters arises mainly because of the recruitment of Notch by Baz to the interface where two siblings touch. In line with our findings in pISC lineage, Notch receptor located basal to the midbody along the pIIa-pIIb interface contributes to the signaling during SOP asymmetric divisions.^23^ Moreover, Baz and Notch form nanoclusters at the pIIa-pIIb interface to aid Notch activation in the pIIa,^72^ supporting the idea that Baz is directly involved in the activation of Notch in the cells where it is located.

We noticed that about half of the *pros*^−/−^ MARCM clones were composed of four cells (Figure S4E), suggesting that a large proportion of the pISC basal daughters divided only once and did not give rise to four cells as did ISC-like cells. We speculate that in the complete absence of Pros expression, many basal daughters did not acquire the ISC-like state. It is possible that a pISC basal daughter with a low level of Pros expression is more likely to have a pISC developmental pattern. Intriguingly, a low-level pulse of nuclear Pros maintains the NB in quiescence,^73^ which again suggests the importance of Pros protein levels.

Mammalian intestinal Prox1^+^ ee cells are converted into ISCs after injury-induced regeneration,^74–76^ comparable with our pISC basal daughter converting process. Mammalian Lgr5^+^ ISC number is affected by inflammation that regulates asymmetric divisions.^77^ Recently, a tumor-suppressive activity of Par-3 in skin cancer has been found,^78^ revealing that Par-3 inhibits cancer development and tumor metastasis.^79^ Therefore, our apical-basal polarity paradigm in pISC lineage might help in understanding the mechanisms of ISC number control and cancer formation in mammalian studies.

### Limitations of the study

In this study, we show that Baz is indispensable for the localization and activation of Notch in apical pISCs. However, we do not know whether Baz enhances Notch localization by binding directly to Notch. It is also possible that Baz recruits Notch to the same complex cluster mediated by phase separation.^80^ The mechanism by which Baz activates Notch signaling should be explored in the future.

## STAR★METHODS

### RESOURCE AVAILABILITY

#### Lead contact

Further information and requests for resources and reagents should be directed to and will be fulfilled by the lead contact, Zheng Guo (guozheng@hust.edu.cn).

#### Materials availability

All plasmids and fly lines generated in this study are available upon request to the lead contact.

#### Data and code availability

The data generated in this study are available upon reasonable request from the lead contact.

This paper does not report original code.

Any additional information required to reanalyze the data reported in this work paper is available from the lead contact upon request.

### EXPERIMENTAL MODEL AND SUBJECT DETAILS

#### *Drosophila* strains and maintenance

Standard cornmeal food was prepared according to the following recipe: 210 g dry inactivated yeast, 900 g yellow cornmeal, 120 g soy flour, 100 g agar (Biosharp), 800 mL light corn syrup, 150 mL propionic acid and 12 L water. Flies were maintained at 25°C and 65% humidity on a 12 h light/dark cycle, unless otherwise indicated. Only female flies were used in all our experiments. The developmental stage or time of development at which analysis were done is indicated in the STAR Methods section. *Drosophila* strains used in this study and their origin are in the key resources table.

### METHOD DETAILS

#### Pros::GFP construction

*Pros*::*GFP* (GFP tagged at the C terminal of prospero) was constructed using a CRISPR/Cas9 mediated homologous recombination method. Two Cas9 targeting sites (Pros-Cas9–1 = GCC CAA TTT TTT AGA GCA GCt gg and Pros-Cas9–2 = ccg ACG CTG CCA TTG CCG CAT CC) were selected near the stop codon of prospero to induce double-strand breaks (DSBs).

To induce homologue based integration and the plasmid cutting by the Cas9 vector, a Pros-Hom-GFP plasmid carrying a GFP at the C-terminal of *pros* with two flanked homologue arms (~1.7 k and ~1.9 k respectively) was constructed, as well as the two non-sense mutations on the Cas9 targeting sites (Pros-Cas9–1 = GCC CAA TTT TTT AGA GCA G (G-T) Ct gg and Pros-Cas9–2 = c(c-t)cg ACG CTG CCA TTG CCG CAT CC), as follows: the homologue arms were amplified (TOYOBO, Cat# KOD-211) from the fly genome (primer pairs Pros-N-homo-5.2 = ACC TCC CTC CTG CTC TTC A/Pros-C-homo-3.2 = ACT GAC AGA CTG ATT GAC CTA CT (3750 bp) and sub-cloned into pEASY-Blunt to obtain the Pros-Homo plasmid. Then, three pairs of primers with a linker sequence and two Cas9 targeting sites non-sense mutations were used to amplify the N terminal homologue arm (Pros-1–5 = cta cgc atc tgc agA GAA TTC TAC T/Pros-1–3 ACT TCC AGA TGC TCT AAA AAA TTG GGC GAC TTG), the GFP tag (GFP-2–5 = TTA GAG CAT CTG GAA GTG **AGC AAG** GGC GAG GAG CTG TTC/GFP-2–3 seg TTA CAT AAA TAA CAG ACC TTG TAC AGC TCA TCC ATG CCC AGG), and the C terminal homologue arm (Pros-3–5 = TAA GTG GAG GAG TTG GCG CTG TCG ACG CTG CCA TTG CCG CAT CCA CTG/Pros-3–3 = TTA CAT AAA TAA CAG ACT AGT GTG TGT GTG TTT GTG GTG TG). The three segments were assembled into the Pros-Homo plasmid by replacing the sequences between EcoRI and SpeI sites on Pros-Homo plasmid using the multi-site clone Kit (Vezeyme, Cat# C113–02).

To obtain two single guide RNA vectors (Pros-guide-1 and Pros-guide-2), two pairs of primers with targeting sites were synthesized (Pros-Cas9–1 = gtc gGC CCA ATT TTT TAG AGC AGC/Pros-Cas9–1R = aaa cGC TGC TCT AAA AAA TTG GGC and Pros-Cas9–2R = gtc gGG ATG CGG CAA TGG CAG CGT/Pros-Cas9–2F = aaa cAC GCT GCC ATT GCC GCA TCC); after annealing, two guide RNAs were subcloned into single guide RNA (sgRNA) vector (modified PMD18T, from Haiyang Chen’s lab), which was digested using BbsI. To assemble the two sgRNAs into the PCR8 vector, two pairs of primers with adaptor sequences (BsaI-U63-F1 = ATG Cgg tctc CTG ACG CTC ACC TGT GAT TGC TC/BsaI-SgRNA-R1 = ATG Cgg tct cGT AAT AAA AAA AGC ACC GAC TCG GTG C and BsaI-U63-F2 = ATG Cgg tct AAT TAA GTC TGT CTT TCC CCT TTC CGC TCA CCT GTG ATT GCT C/BsaI-SgRNA-R4 = ATG Cgg tct cGG AGT AAA AAA AGC ACC GAC TCG GTG C) were used to amplify the two guide RNAs, and assembled through the golden gate mix (NEB). The sgRNAs (PCR8-Pros-guide1/2) was then exchanged to the pUAST-attb vector through attP/AttB recombination (Invitrogen Gateway LR Clonase Enzyme Mix, Cat# 11791019) to obtain the pUAST-AttB-Pros-guide1/2.

The pUAST-AttB-Pros-guide1/2 was integrated into the 51D site by microinjection (performed by Unihuaii. Ltd) to obtain the *pros* guide RNA transgenic fly (*Pros-guide-RNA*). The *Pros-guide RNA* transgenic fly was crossed with yw; nos-Cas9 (II-attP40) to induce DSBs. The F1 embryos with DSBs were injected with Pros-Hom-GFP plasmid. After eclosion, they were single crossed with yw122; If/CyO; MKRS/TM6B flies of the opposite sex. The F2 male flies were single crossed with yw122; If/CyO; MKRS/TM6B, and the recombination events were verified with PCR (Prospero-N homo 3.2 = CTG GAG TTG GTG GTG GTA GCG GT/Pros-1–5 = gat ttt caa aat gaa taa cta aag tg, 1127 bp) and immunostaining of Pros and GFP.

#### *UAS-mira* construction

*Miranda* cDNA was amplified from the BL#56555 *UAS-mira*::*GFP* genome (primer pairs: Mira-N = ATG TCT TTC TCC AAG GCC AA/Mira-C= CTA GAT GTT GCG CGC CTT GA, 2490bp) and sub-cloned into pEASY-Blunt to obtain the Mira cDNA plasmid. Mira cDNA with homologues linker of pUAST (Mira-F = tcg tta aca gat ctg cgg cc ATG TCT TTC TCC AAG GCC AA/mira-R = caa aga tcc tct aga ggt ac CTA GAT GTT GCG CGC CTT GA) was assembled into the pUAST-attB plasmid by replacing the sequences between NotI and KpnI using ClonExpress II One Step Cloning Kit (Vezeyme, Cat# C112–01). pUAST-attB-*mira* was integrated to the 51D site by microinjection to obtain the *UAS-mira* transgenic fly (performed by Unihuaii. Ltd). *FRT19A*; *UAS-mira* was made to allow MARCM analysis.

#### Baz antibody production

The N-terminal (amino acids 1–297) portion of Baz was fused with GST tag and Baz was cloned into pEGX-6P-1 expression vectors, then used to transform BL21A competent cells according to the method of Wodarz A et al..^11^ GST-tagged protein was purified from the lysate using GST purification kit and was injected into a rabbit (Wuhan Proteinab Biotech Co.,Ltd). The serum was collected over a period of 3 months with 5 injections of immunization. Baz antibody was purified by affinity purification (Wuhan Proteinab Biotech Co.,Ltd) and tested for specificity by Western blot analysis and immunostaining.

#### Live imaging of pupal midgut

##### Prepare live imaging buffer (LIB)

8.5 mL Schneider’s *Drosophila* Medium (Gibco, Thermo Fisher Scientific, Cat# 21720024) was supplemented with 1.5 mL FBS (Gibco, Thermo Fisher Scientific. Cat# 10091148) and 2 mg human insulin (Sigma, Cat# I0305000), and pH was adjusted to 7.0.

##### Prepare live imaging gel (LIG)

0.5 g gelatin (Sigma-Aldrich, Cat# G2500) was added into 5 mL of LIB, then heated at 50°C to melt the gel. Both LIB and LIG were divided into 500 μL aliquots and stored at 4°C for up to 1 week. Aliquots of LIG were heated at 37°C before experiments.

##### Preparation of pupal guts for live imaging

Two pieces of cover glass with a size of 10 × 22 mm were attached to a lumox dish 50 (Sarstedt, Cat# 15090935) using LIG, with ~1 cm gap in between. Intact pupal guts were dissected in LIB and transferred on a 22 × 22 mm cover glass. The excess LIB was carefully removed using filter paper. An amount of 80 μL LIG at 37°C was dropped into the 1 cm-gap, then the 22 × 22mm cover glass was quickly placed on the top of 10 × 22 mm cover glasses, covering the guts by LIG without the formation of air bubbles. After LIG was cooled down and stabilized, the cover glasses were finally sealed using Halocarbon oil 27 (Sigma-Aldrich, Cat# H8773) to avoid evaporation.

##### Setting of time-lapse experiments on confocal microscopy

GFP and tdTomato signals were imaged on a Zeiss LSM 800 confocal microscope equipped with a Plan-Apochromat 63x/1.40 Oil DIC M27 objectives (imaging medium: immersol 518 F obtained from Zeiss). Zeiss Definite Focus.2 was used to avoid focus drift. Time-lapse Images were acquired by ZEN 2.1 with Time Lapse Module. A total of 8–12 μm z stack images (2 μm intervals) were acquired at 512 × 512 pixels (0.198 μm × 0.198 μm) every 60 s at room temperature (25°C) for over 3 h, with a pixel time of 1.03 μs and frame averaging of 2% Laser strength, pinhole and other settings were fixed for all the time-lapse experiments.

#### Immunostaining and fluorescence microscopy

Samples were dissected in 1x PBS, and fixed in 4% formaldehyde for 2 h. Primary antibodies were the following and used at the following dilutions: mouse anti-Pros at 1:100 (MR1A, Developmental Studies Hybridoma Bank); chicken anti-GFP at 1:10,000 (Abcam, Ab13970); rabbit anti-GFP at 1:10,000 (Abcam, Ab6556); rat anti-α-tubulin at 1:2,000 (Abd Serotec MCA78G); rabbit anti-Mira at 1:1,000 (antigen: peptide 96C, from amino acid 96 to 118, A96C, a kind gift from Prof. Yuh Nung Jan, University of California School of Medicine, San Francisco); rabbit anti-GST-Baz Nterm at 1:2,000 (final bleed, a kind gift from Elisabeth Knust, Max-Planck-Institute of Molecular Cell biology and Genetics, Dresden); rabbit anti-Baz at 1:10,000 (this paper); anti-PKCζ (C20; 1:1,000; Santa Cruz Biotechnology, Inc. A kind gift from Prof. Jiong Chen, Nanjing University); rabbit anti-phospho-histone H3 at 1:10,000 (H3ser10, Millipore #2465253); chicken anti-β-galactosidase at 1:10,000 (Abcam, Ab9361); mouse anti-Notch-intra at 1:100 (C17.9C6, Developmental Studies Hybridoma Bank). The Alexa Fluor–conjugated secondary antibodies were used at 1:4,000 (Molecular Probes and Invitrogen). Guts were stained with 1 μg/mL DAPI (Sigma-Aldrich), mounted in 70% glycerol, and imaged with a confocal microscope (Zeiss LSM800) using Plan-Apochromat 20x/0.8 M27, 63x/1.40 Oil DIC M27 objectives (imaging medium: immersol 518 F obtained from Zeiss). The imaging temperature was the room temperature (25°C). The acquisition and processing software used was ZEN 2.1. Images were processed in Photoshop CC (Abode) and Illustrator CS6 (Adobe) for image merging and resizing.

#### MARCM clone analysis during the pupal period

Flies were cultured in a 25°C incubator, Heat-shock were conducted at LL3 (*mira* RNAi and *mria* mutant), or 0 h APF (*numb mutant*, *pros mutant*, *baz mutant*, *par-6 mutant*, *aPKC mutant*, *baz par-6* double *mutant*, *par-6* RNAi *and aPKC* RNAi), or 24 h APF (*mira* overexpression, *mria* mutant, and *Notch* mutant) for 1 h at 30°C and then moved back to 25°C, dissect at specified time of (50 h, 60 h or 90 h APF) and immunostaining. White pupae were considered as 0 h APF. For LL3 heat shock, LL3 female larvae were selected and heat shocked, then cultured at 25°C, collect while pupa into a new vial designated as 0 h APF.

The benefit of MARCM induction at LL3 is better eliminating RNA or protein perdurance in pISC lineage, but the consequence is that MARCM clones would label enterocytes,^52^ thus making difficult to evaluate single pISC MARCM clones. Vice versa, MARCM clones induced at 0 h or 24 h APF are easy to identify, but it is hard to remove the RNA or protein perdurance (depending on the nature of genes), especially for those pISC clones located in the anterior pupal midguts, which enters asymmetric divisions quicker than the posterior clones after clone induction.

#### *Notch* knockdown during pupal development

*esg*^*ts*^*>GFP* (control) and *esg*^*ts*^*>GFP UAS-Notch RNAi* flies were cultured at 18°C. LL3 female larvae were placed into new vials with fresh cornmeal food. Samples were transferred to 30°C to activate the *Gal4* function. Flies were then dissected at 41 h (Figure 1N), 46 h (Figures 1O and 1P), 52 h (Figure 1Q) and 72 h APF at 30°C (Figure 1R), and subjected to immunofluorescent staining. Based on previous studies comparing pupal development at 18°C and 30°C–25°C,^81^ the chosen dissection times roughly corresponded to 46 h, 52 h, 60 h, and 90 h APF at 25°C.

#### Ectopic expression of Mira during pupal development

*esg*^*ts*^*>GFP* (*Control*) and *esg*^*ts*^*>GFP UAS-mira* flies were cultured at 18°C. LL3 female larvae were placed into new vials with fresh cornmeal food. When LL3 female larvae started the metamorphosis, white pupae were placed into an empty vial and considered as 0 h APF. Samples were kept at 18°C until 78 h APF and then they were transferred to 30°C to activate the *Gal4* function. Based on previous studies comparing pupal development at 18°C and 30°C–25°C, the activation times roughly correspond to 36 h APF at 25°C. Dissections were performed before eclosion, based on the appearance of the black wings and mature bristles, and subjected to immunofluorescent staining.

#### Ectopic expression of Mira::GFP and Mira by *esg-Gal4* during pupal development

*yw*; *NP5130* (*esg-Gal4*) (*Control*) and *esg>UAS-mira*::*GFP or UAS-mira* flies were cultured at 25°C. LL3 female larvae were placed into new vials with fresh cornmeal food. When LL3 female larvae started the metamorphosis, white pupae were placed into an empty vial and considered as 0 h APF. Flies were dissected at 46 h, 52 h, 60 h or 90 h APF, and subjected to immunofluorescent staining.

#### *mira* knockdown during pupal development

*esg*^*ts*^*>GFP* (*Control*) and *esg*^*ts*^*>GFP UAS-mira RNAi* flies were cultured at 18°C. LL3 female larvae were placed into new vials with fresh cornmeal food and then they were transferred to 30°C to induce *esg-Gal4* expression. White pupae were placed into an empty vial and considered as 0 h APF. Flies were kept at 30°C and then dissected at 41 h APF (corresponding to 46 h APF at 25°C, ISC mitotic division), 46 h APF (corresponding to 52 h APF at 25°C, ISC-like cell mitotic division), 50 h APF (corresponding to 56 h APF at 25°C, EMC mitotic division) or 72h APF (corresponding to 90 h APF at 25°C), and subjected to immunofluorescent staining.

#### *pros* knockdown during pupal development

*esg*^*ts*^*>GFP* (control) and *esg*^*ts*^*>GFP UAS-pros RNAi* flies were cultured at 18°C. White female pupae were transferred to 30 °C at 0h APF to activate the *Gal4* function. Flies were then dissected at 43 h APF, and subjected to immunofluorescent staining.

#### Ectopic expression of pros during pupal development

*esg*^*ts*^*>GFP* (control) and *esg*^*ts*^*>GFP UAS-pros* flies were cultured at 18°C. LL3 female larvae were placed into new vials with fresh cornmeal food. When LL3 female larvae started the metamorphosis, white pupae were placed into an empty vial and considered as 0 h APF. Flies were transferred to 30°C or 25°C to induce *esg-Gal4* expression. Flies were then dissected at 43 h APF and 30°C and 50 h APF and 25°C and subjected to immunofluorescent staining.

#### *Baz*, *Par-6* and *aPKC* knockdown, and ectopic expression of *aPKC-CAAX* and *aPKC-K*D during pupal development

*esg*^*ts*^*>GFP* (control), *esg*^*ts*^*>GFP UAS-baz RNAi*, *esg*^*ts*^*>GFP UAS-par-6 RNAi*, *esg*^*ts*^*>GFP UAS-aPKC RNAi*, *esg*^*ts*^*>GFP UAS-aPKC-CAAX* and *esg*^*ts*^*>GFP UAS-aPKC-KD* flies were cultured at 18°C. LL3 female larvae were placed into new vials with fresh cornmeal food and then they were transferred to 30°C to induce *esg-Gal4* expression. White pupae were placed into an empty vial and considered as 0 h APF. Flies were then dissected at 41 h, 43 h, 46 h, 47 h, 52 h, 72 h APF at 30°C and subjected to immunofluorescent staining.

#### *Cdk2*, *geminin* and *Cdk1* knockdown during pupal development

*esg*^*ts*^*>GFP* (control), *esg*^*ts*^*>GFP*, *Cdk2 RNAi*, *esg*^*ts*^*>GFP*, *geminin RNAi*, and *esg*^*ts*^*>GFP*, *Cdk1 RNAi* flies were cultured at 18°C. LL3 female larvae were placed into new vials with fresh cornmeal food. Samples were transferred to 30°C to activate the *Gal4* function at 0 h APF. *esg*^*ts*^*>GFP*, *Cdk2 RNAi*, *geminin RNAi* and part of *Cdk1 RNAi* flies were then dissected at 43 h APF (corresponding to 46 h APF at 25°C), and subjected to immunofluorescent staining. Some *Cdk1 RNAi* flies were transferred to 18°C to recover for 8 h and then dissected and subjected to immunofluorescent staining. The remaining *Cdk1 RNAi* flies were kept at 30°C until reaching 47 h APF and transferred to 18°C to recover for 8 h, then dissected and subjected to immunofluorescent staining.

#### *Cdk2*, *geminin* and *Cdk1* knockdown combined with the Fucci system

*UAS-GFP-E2F1*,*UAS-RFP-nls-CycB* were recombined with the RNAi stocks and crossed with *esg-Gal4*; *tub-Gal80*^*ts*^ stock, then flies were cultured at 18°C. White female pupae were transferred to 30 °C at 0 h APF to activate the *Gal4* function. Flies were then dissected at 43 h APF, and subjected to immunofluorescent staining.

#### Other RNAi knockdown during pupal development

For other RNAi knockdown during pupal development, LL3 female larvae were placed into new vials with fresh cornmeal food. Samples were transferred to 30°C to activate the *Gal4* function at 0 h APF and dissected at 66 h APF for immunofluorescent staining.

### QUANTIFICATION AND STATISTICAL ANALYSIS

#### Pros fluorescence quantification

In each experiment comparing protein intensity, the flies in the control group and experimental groups were dissected, fixed, and immuno-stained at the same condition. Confocal images were taken under the same settings. The Pros intensity of the control group was evaluated with repeated before the experiment groups. No significant difference was found between control repeats. Prospero fluorescent quantification was measured using the mean gray value function in ImageJ. Fluorescent quantification was performed by selecting a ROI (the area of ISC lineage cell) in the raw confocal images, measuring the mean intensity of that area, and subtracting the mean fluorescent background (at least 3 areas next to the cell were quantified). The intensity of Pros was the product of cell area and corrected mean intensity. The average intensity of Pros staining (AIPS) at metaphase of pISC is considered as 100.

#### Pros::GFP Fluorescence quantification

The fluorescence quantification of each cell was manually performed by Fiji ImageJ for every frame during the entire timeline. While cell shapes were dynamically changed during mitotic divisions, the region of interest (ROI) was carefully depicted based on the area of the cell. The fluorescence intensity of ROIs in the green channel (from Pros:GFP) was determined in projection images of recorded z-stacks, where the background intensity was excluded. The mean intensity of the background was determined by selecting three individual regions next to the ROIs. The heatmap was performed in GraphPad Prism 8.

#### *Pros*-LacZ, *NRE*-LacZ and aPKC fluorescence quantification

Flies in control group and experimental groups were dissected, fixed, and immuno-stained at the same condition. Confocal images were taken under the same settings. *pros*-lacZ, *NRE*-LacZ and aPKC fluorescent quantification were measured using the mean gray value function in ImageJ. Fluorescent quantification was performed by selecting a ROI (the area of ISC lineage cell) in the raw confocal images, measuring the mean intensity of that area, and subtracting the mean fluorescent background (at least 3 areas next to the cell were quantified).

#### Baz:Pros ratio quantification

Flies in control group and experimental groups were dissected, fixed, and immuno-stained at the same condition. Confocal images were taken under the same settings. Baz and Pros fluorescent quantification were measured using Zesiss ZEN 3.4 by selecting a ROI (the area of ISC lineage cell) in the raw confocal images, measuring the mean intensity of that area, and subtracting the mean fluorescent background (at least 3 areas next to the cell were quantified), the average Baz: Pros ratio in ISCP1 were defined as 1.0.

#### Statistics of the proportion of ISC to enteroendocrine cell

The ratio of ISC cells to ee cells was calculated by counting the number of Pros^−^ GFP^+^ cells divided by the Pros^+^ cells.

#### Localization analysis of the Baz and Notch receptor

Flies of *esg*^*ts*^*>GFP* (control), *esg*^*ts*^*>baz RNAi* and *esg*^*ts*^*>aPKC-CAAX* were dissected, fixed, and immuno-stained at the same condition. Confocal images were taken under the same settings. Baz and Notch fluorescence distribution were measured using Zeiss ZEN 3.4 by drawing a line of interest (white bar in Figures 6F–6H) in the raw confocal images. Data were exported to excel and the graphics were performed in GraphPad Prism 8.

#### Statistical analysis

Statistical analysis was performed using Prism (GraphPad Software). Student t-test was used to compare two groups, while one-way analysis of variance (ANOVA) was used to compare multiple groups. Radial histogram graph was made using OriginPro 8.5.1. (OriginLabs). Results were expressed as mean ± Standard Deviation (SD). All of the statistical details of experiments can be found in the figures and figure legends.

## Supplementary Material

supplemental2

supplemental1

supplemental3

supplemental4

supplemental6

supplemental5

supplemental7

supplemental9

suppemental8

supplemental10

Supplemental information can be found online at https://doi.org/10.1016/j.celrep.2023.112093.

## Figures and Tables

**KEY RESOURCES TABLE:** 

REAGENT or RESOURCE	SOURCE	IDENTIFIER

Antibodies		

Rabbit anti-Baz	This paper	N/A
Rabbit anti-Baz	Wodarz A; Nature. 1999	Cat# baz, RRID:AB_2570125
Chicken anti-GFP	Abcam	Cat# 13970; RRID:AB_300798
Rabbit anti-GFP	Abcam	Cat# 6556; RRID:AB_305564
Mouse anti-Pros	DSHB	Cat# MR1A; RRID: AB_528440
Mouse anti NICD	DSHB	Cat# C17.9C6; RRID: AB_528410
Rabbit anti-Mira	Shen CP; Cell. 1997	Cat# mira; RRID:AB_2569537
Rabbit anti-α-tubulin	Abcam	Cat# 52866; RRID:AB_869989
Chicken anti-β-galactosidase	Abcam	Cat# 9361; RRID:AB_307210
Rabbit anti-PH3	Millipore	Cat# 06–570; RRID:AB_310177
Rabbit anti-PKCζ	Santa Cruz Biotechnology	Cat# sc-17781; RRID:AB_628148

Chemicals, peptides, and recombinant proteins		

4% formaldehyde	Sigma-Aldrich	F8775
DAPI	Sigma-Aldrich	D9542
Schneider's *Drosophila* Medium	Gibco, Thermo Fisher Scientific	21720024
FBS	Gibco, Thermo Fisher Scientific	10091148
Insulin	Sigma-Aldrich	I0305000
Gelatin	Sigma-Aldrich	G2500
Halocarbon oil 27	Sigma-Aldrich	H8773
lumox^®^ dish 50	Sarstedt	15090935

Experimental models: Organisms/strains		

*pros::GFP* (*III*)	This paper	N/A
*UAS-mira* (*II*)	This paper	N/A
*UAS-mira::GFP* (*II*)	Bloomington	BL56555
*hs-flp*	Benjamin Ohlstein	N/A
*esg-Gal4* (*II*)	Benjamin Ohlstein	N/A
*Tub-Gal4* (*III*)	Benjamin Ohlstein	N/A
*Tub-Gal80^ts^* (*II*)	Benjamin Ohlstein	N/A
*UAS-GFP* (*II*)	Benjamin Ohlstein	N/A
*NRE-lacZ* (*X*)	Benjamin Ohlstein	N/A
*esg-Gal4 UAS-GFP/CyO*	Benjamin Ohlstein	N/A
*esg-Gal4 UAS-GFP tub-Gal80/CyO*	Benjamin Ohlstein	N/A
*yw; NP5130 (esg-Gal4)*	Ken Irvine	N/A
*yw hs-Flp; esg-Gal4 10XUAS-Myr:tdTomato; pros::GFP/TM6B Tb*	This paper	N/A
*yw hs-Flp; esg-Gal4 tub-Gal80^ts^ 10XUAS-Myr:tdTomato;pros::GFP/TM6B Tb*	This paper	N/A
*10×UAS-Myr:tdTomato* (*II*)	Ken Irvine	N/A
*Notch^55e11^FRT19A/FM7*	Bloomington	BL28813
*FRT19A; ry506*	Bloomington	BL1709
*MARCM 19A*	Gary Struhl	N/A
*FRT 40A*	Benjamin Ohlstein	N/A
*MARCM40A*	Benjamin Ohlstein	N/A
*FRT42D*	Benjamin Ohlstein	N/A
*MARCM42D*	Benjamin Ohlstein	N/A
*FRT 82B*	Benjamin Ohlstein	N/A
*MARCM 82B*	Gary Struhl	N/A
*UAS-Notch RNAi* (*III*)	Bloomington	BL7078
*UAS-Notch^ECN^* (*II*)	Bloomington	BL#51667
*UAS-mira::GFP* (*II*)	Bloomington	BL56555
*FRT82B, mira^L44^*	Michael O'Connor	N/A
*FRT82B, mira^ZZ176^*	Michael O'Connor	N/A
*mira RNAi* (*III*)	Tsinghua Fly Center	THU0809
*baz RNAi* (*III*)	Bloomington	31523
*baz RNAi* (*III*)	Tsinghua Fly Center	THU1861
*UAS-par-6 RNAi* (*III*)	Tsinghua Fly Center	THU3865
*UAS-aPKC RNAi* (*III*)	Tsinghua Fly Center	THU5841
*UAS-aPKC-CAAX* (*II*)	Huang Jianhua	N/A
*UAS-aPKC-KD*	Huang Jianhua	N/A
*FRT40A, numb^1^*	Gary Struhl	BL4096
*FRT40A, numb^2^*	Gary Struhl	N/A
*par-6^Δ226^, FRT19A*	Bloomington	BL81043
*par-6^29vv^, FRT19A*	Bloomington	BL81042
*baz^FA50^, par-6^Δ226^, FRT19A*	Bloomington	BL81046
*FRT42D,aPKC^k06403^*	Kyoto	BL111194
*FRT19A, baz^4^/TM7B*	Chris Doe	N/A
*UAS-pros RNAi* (*III*)	Tsinghua Fly Center	THU2372
*UAS-3XFLAG-pros.S* (*X*)	Bloomington	BL32245
*FRT82B, pros^17^*	Benjamin Ohlstein	N/A
*UAS-Cdk2 RNAi* (*II*)	Tsinghua Fly Center	TH201500107.s
*UAS-geminin RNAi* (*II*)	Tsinghua Fly Center	THU5691
*UAS-Cdk1 RNAi* (*III*)	Tsinghua Fly Center	THU5882
*pros-lacZ/TM3.ser*	Bloomington	BL11174
*UAS-GFP-E2F1,UAS-RFP-nls-CycB/CyO; MKRS/TM6B.Tb*	Bloomington	BL55110
*UAS-GFP-E2F1,UAS-RFP-nls-CycB/TM6B.Tb*	Bloomington	BL55111
*numb::GFP*	Schweisguth Lab	N/A
*UAS-ttk RNAi* (*II*)	Tsinghua Fly Center	TH201501029.S
*UAS-phyl RNAi* (*III*)	Tsinghua Fly Center	THU3242

Software and algorithms		

ImageJ	NIH	http://adm.irbbarcelona.org/image-j-fiji
Photoshop CC	Adobe	http://www.adobe.com
Photoshop Illustrator CS6	Adobe	http://www.adobe.com
Prism 6	GraphPad	https://www.graphpad.com/
OriginPro	OriginLabs	https://www.originlab.com/

Other		

Zeiss LSM 800 confocal microscope	Zeiss Microsystems	N/A
20 × 0.8 NA objective	Zeiss Microsystems	N/A
63 × 1.4 NA oil-immersion objective	Zeiss Microsystems	N/A

## Highlights

- Apical Baz inhibits Pros accumulation by activating Notch
- Cell polarity defects in pISCs result in reduced basal allocation of Pros
- Lacking a threshold Pros level, pISC basal daughters become ISC-like cells
- Over-threshold Pros inhibits *mira* transcription, thus leading to symmetric division

## INCLUSION AND DIVERSITY

We support inclusive, diverse, and equitable conduct of research.
