# Supplementary material for: Apical-basal polarity precisely determines intestinal stem cell number by regulating Prospero threshold
Source: Cell Rep. Author manuscript; Available in PMC 2026 Feb 23. (PMC12927587; doi:10.1016/j.celrep.2023.112093)
Supplement: supplemental1 [file NIHMS2135711-supplement-supplemental1.pdf]

**Cell Reports, Volume *42***

## Supplemental information

# Apical-basal polarity precisely determines intestinal stem cell number by regulating Prospero threshold

**Song Wu, Yang Yang, Ruizhi Tang, Song Zhang, Peizhong Qin, Rong Lin, Neus Rafel, Elena M. Lucchetta, Benjamin Ohlstein, and Zheng Guo**

## Supplemental information

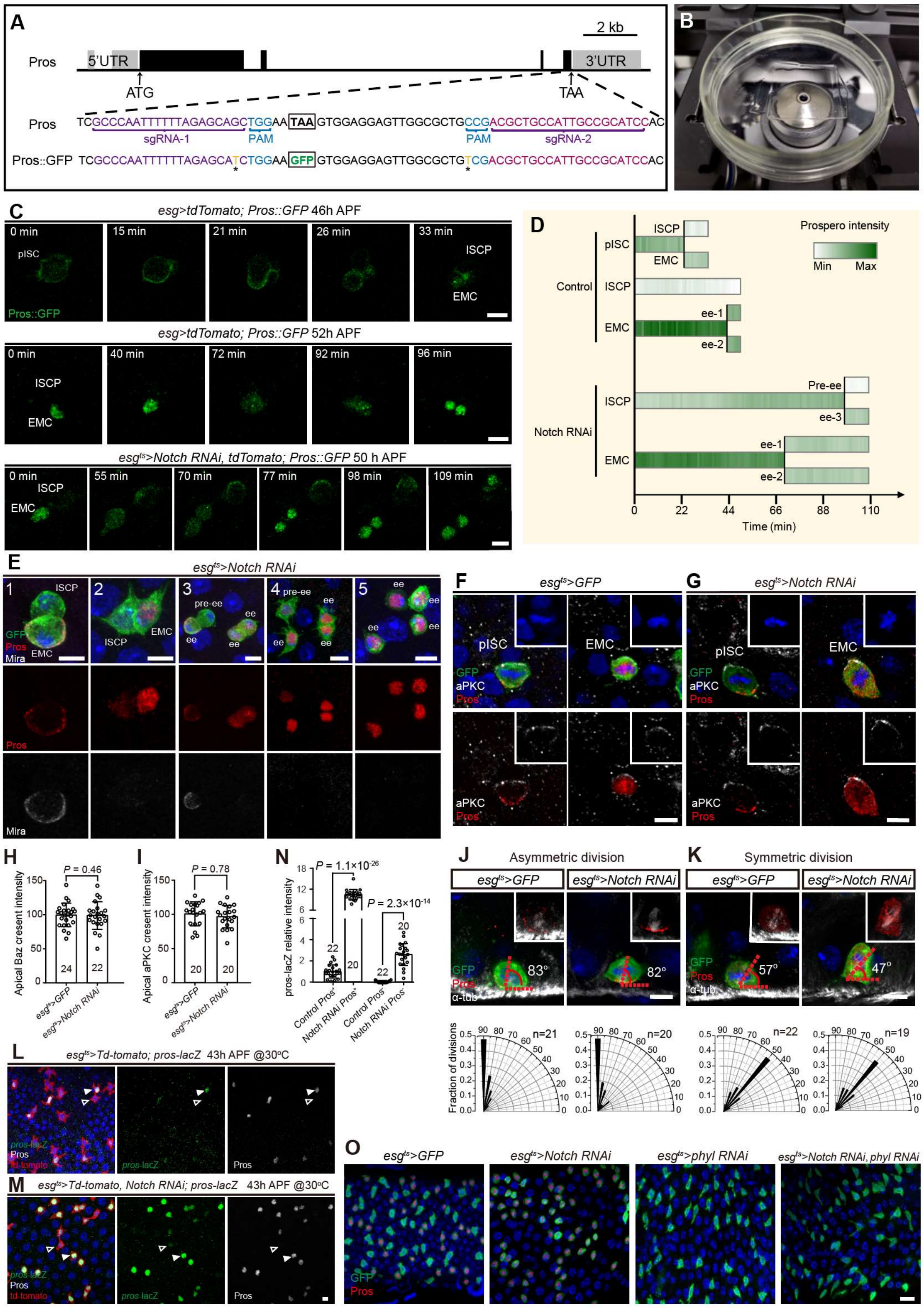

**Supplementary Figure 1. Asymmetrical division of *Notch* deficient pISC and *Notch* signaling requirement for inhibiting Pros expression in the apical daughter.**

(A) Schematic representation of Pros::GFP genomic knocking in KI line. Two sgRNAs were used to efficiently induce *pros* 3' DNA homologous recombination. Pros stop codon TAA (in the box) was replaced by GFP. The stars indicate the synonymous mutations blocking further sgRNA-mediated DNA cleavage.

(B) Set up of a device used for pupal midgut *ex vivo* live-imaging on an inverted Zeiss LSM800 confocal microscope.

(C and D) Split Channels of Pros::GFP in Figure 1 h to j (C). Diagrams depicting changes in the intensity of Pros::GFP in *control* and *esg$^{ts}$>Notch RNAi* pISC, ISCP, EMC and their progenies over time according to the frames on c (D). Green represents Pros expression according to the scale (white: low; dark green: high). See also Videos S1, S2 and S3.

(E) Mira and Pros colocalize on the basal daughter cell membrane during cytokinesis of pISC asymmetric division in *esg$^{ts}$>Notch RNAi* pISC lineage (1). After pISC mitotic division, Pros is localized in the nuclei of EMC, while a weak Pros appeared in ISCP (2). After asymmetric division of ISCP (3), three Pros$^{+}$ ee cells and one weak Pros$^{+}$ pre-ee cell was present in the *Notch RNAi* pISC lineage (4). Finally, four progenies of pISC become Pros$^{+}$ ee cells (5).

(F and G) Apical aPKC crescent staining at metaphase of pISC asymmetric division and EMC symmetric division in *esg$^{ts}$>GFP* (F) and *esg$^{ts}$>Notch RNAi* (G) midguts. Inserts show DAPI (blue) and aPKC (white) staining.

(H and I) Quantification of the apical crescent intensity of Baz (H) and aPKC (I) staining at metaphase of *control* (*esg$^{ts}$>GFP*) and *esg$^{ts}$>Notch RNAi* pISCs. The dividing line between apical and basal is the equatorial plate where the chromosomes are arranged at the metaphase phase of the dividing cell. The average intensity of Baz or aPKC staining on the apical crescent was defined as 100. Scored cell number was indicated in the column. Error bar, SD.

(J and K) Representative images and radial histogram quantification of the division angles at metaphase of asymmetric (J) and symmetric divisions (K) in *esg$^{ts}$>GFP* and *esg$^{ts}$>Notch RNAi* pupal midguts. n, scored cell number.

(L and M) *pros*-LacZ and Pros staining in *esg$^{ts}$>Td-tomato* (control) (L) and *esg$^{ts}$>Td-tomato, Notch RNAi* (M) pupal midgut at 43 hours APF.

(N) Quantification of the intensity of *pros*-LacZ staining in control and *Notch RNAi* Td-tomato$^{+}$ Pros$^{+}$ cells and Td-tomato$^{+}$ Pros$^{-}$ cells. the average intensity of *pros*-LacZ staining in control Pros$^{+}$ cells was defined as 1.0. Scored cell number was indicated in the column. Error bar, SD.

(O) *phyl* is epistatic to *Notch* during pISC lineage development. Simultaneously knockdown *Notch* and *phyl* resulted in no ee cell formation, exhibiting the *phyl* knockdown phenotype.

Data are represented as mean ± SD. Scale bars, 5 μm.

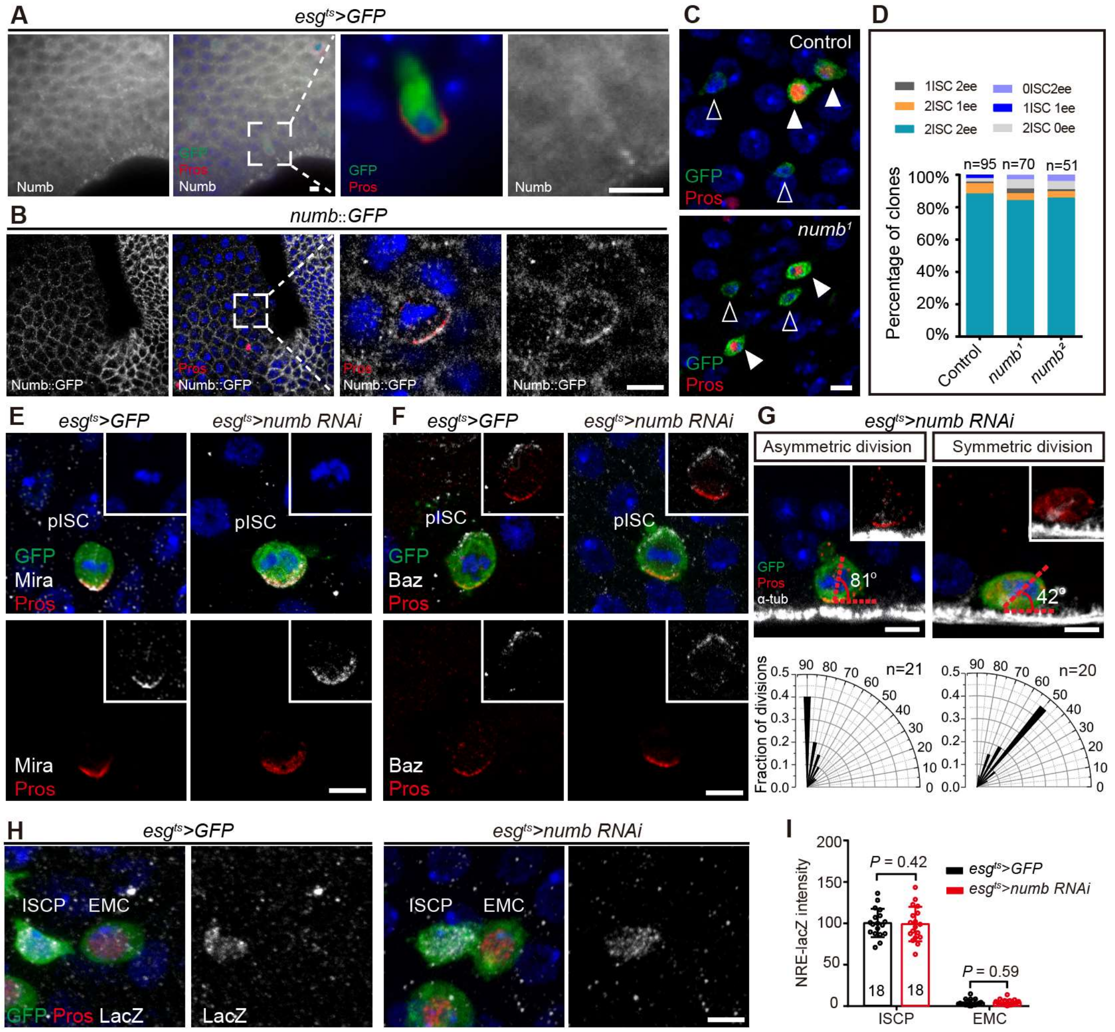


**Supplementary Figure 2. Numb is dispensable in the binary fate decision in pISC lineage.**

(A) Numb antibody staining in *esg^ts>GFP* pupal midgut at 44 h APF.

(B) Basal crescent of Numb::GFP in a Pros$^{+}$ dividing pISC in pupal midgut at 44 h APF.

(C and D) Representative control and *numb^1* MARCM clones at 90 h APF (C). Bar graph representing the percentage of different clone types in control, *numb^1 and numb^2* MARCM clones induced at white pupal and examined at 90 h APF (D). n, scored clone number.

(E) Representative images shown that Mira and Pros basal crescent located in *esg^ts>GFP* and *esg^ts>numb RNAi* dividing pISCs. Observed cell number>20, gut number>5.

(F) Representative images shown that apical Baz crescent and basal Pros crescent located in *esg^ts>GFP* and *esg^ts>numb RNAi* dividing pISCs. Observed cell number>20, gut number>5.

(G) Representative images and radial histogram quantification of the division angles at metaphase of asymmetric and symmetric divisions in *esg^ts>GFP* and *esg^ts>numb RNAi* pupal midguts. n, scored cell number.

(H) The Notch signaling reporter *NRE-lacZ* is present in ISCP in *esg^ts>GFP* and *esg^ts>numb RNAi* animals.

(I) Quantification of the intensity of *NRE*-LacZ staining in sibling daughters after pISC asymmetric divisions in in *esg^ts>GFP* and *esg^ts>numb RNAi* animals. The average intensity of *NRE*-LacZ staining in control ISCP was defined as 100. Scored cell number was indicated in the column. Error bar, SD. Scale bar, 5 μm.

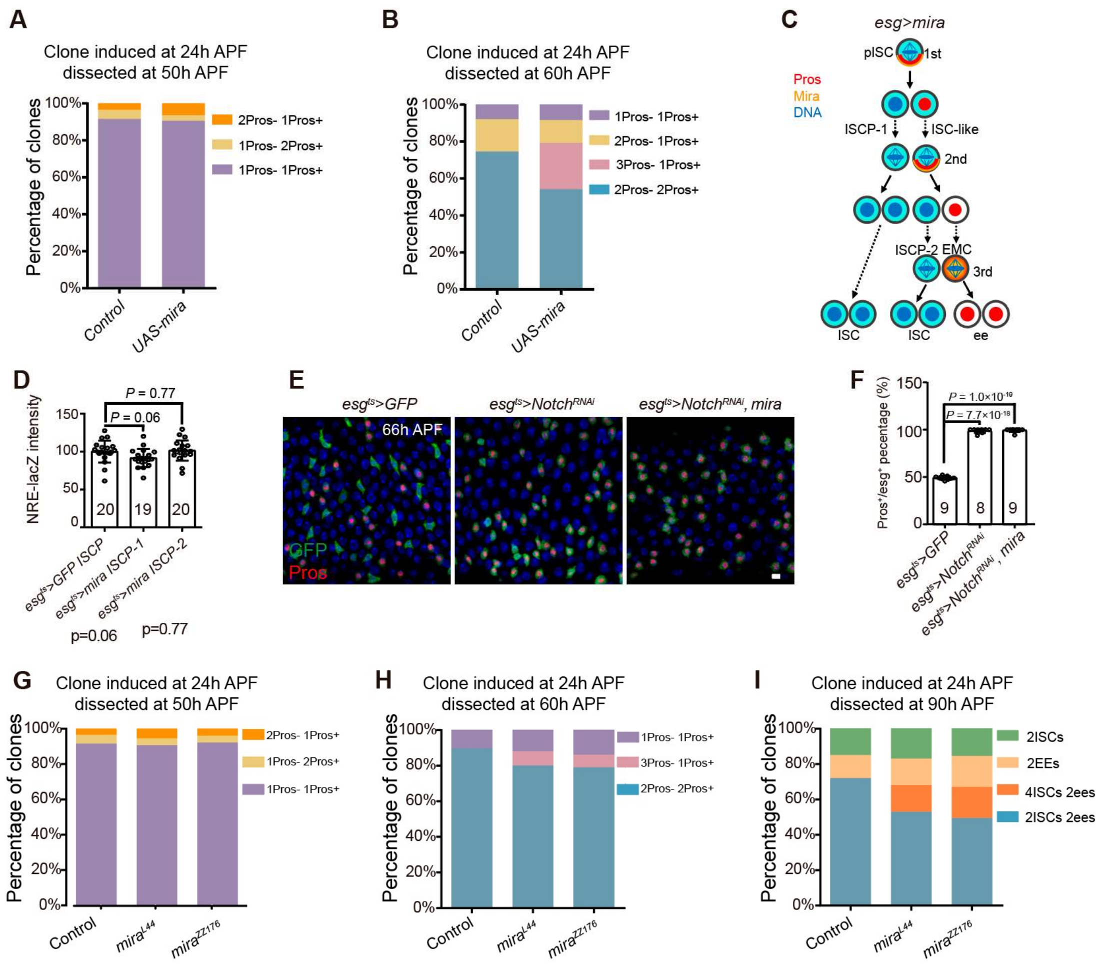

**Supplementary Figure 3. Ectopic *mira* expression or *mira* mutant converts pISC basal daughter to ISC-like cell.**

(A and B) Percentage of clones in control and *UAS-mira* MARCM induced at 24 h APF and examined at 50 h (A), and 60 h APF (B). The clone number was n=72 in the control and n=81 in the *UAS-mira* at 50 h APF (A), n=90 in the control and n=92 in the *UAS-mira* at 60 h APF (B).

(C) Schematic representation of the division of the ISC lineage when Mira was ectopically expressed during the pupal stage.

(D) Quantification of the intensity of *NRE*-LacZ staining in control (*esg$^{ts}$>GFP*) ISCP and *esg$^{ts}$>mira* ISCP-1, ISCP-2. The average intensity of *NRE*-LacZ staining in control ISCP was defined as 100. Scored cell number was indicated in the column. Error bar, SD.

(E) Genetic interactions between *Notch* knockdown and overexpression of Mira. Pupal midguts were dissected at 66 h APF. Overexpression Mira and knockdown Notch together showed the Notch knockdown phenotype.

(F) Statistics of Pros$^+$/esg$^+$ cells ratio referred to (E). The ratio was calculated by counting cells in a 20X (objective) image of posterior pupal midgut. Number of counted images was indicated in the column. Error bar, SD.

(G to I) Percentage of clones in the control and *mira* mutant MARCM induced at 24 h APF and examined at 50 h (G), 60 h (H), and 90 h APF (I). The number of clones was n=36 in the control, n=38 in *mira$^{L44}$* and n=32 in *mira$^{ZZ176}$* at 50 h APF, while it was n=62 in the control, n=70 in *mira$^{L44}$* and n=68 in *mira$^{ZZ176}$* at 60 h APF. Finally, the number of clones was n=46 in the control, n=114 in *mira$^{L44}$*, and n=104 in *mira$^{ZZ176}$* at

90 h APF.

Scale bars, 5 μm.

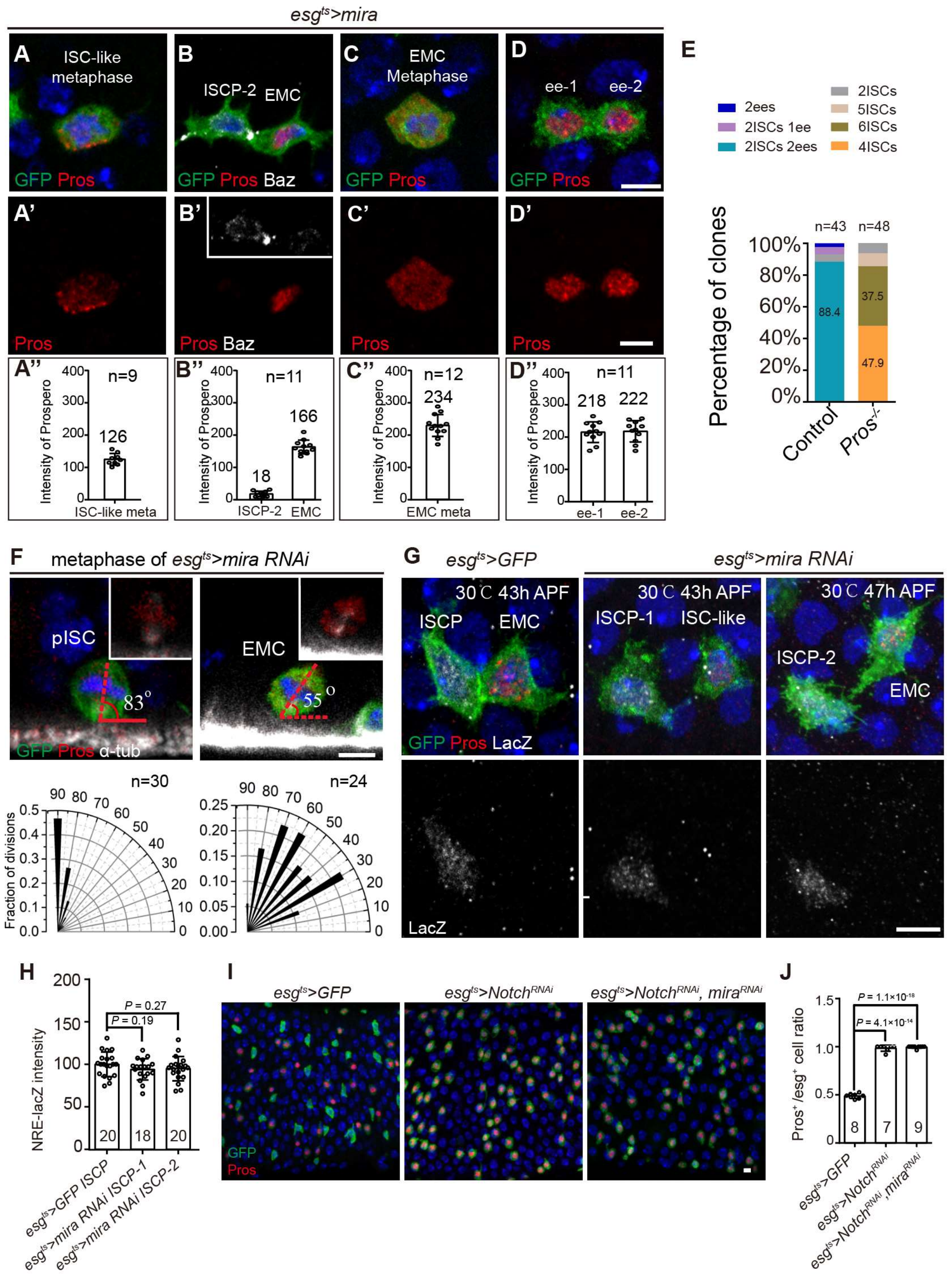


**Supplementary Figure 4. pISC basal daughters become ISC-like cells without a threshold Pros level.**

(A to D) Representative images and quantification of the intensity of Pros staining in ISC lineage after *mira* overexpression at 30°C. The insert in b' show Baz staining (white). Error bar, SD.

(E) Percentage of clones in Control and *control* and *pros*$^{17}$ MARCM clones induced at 0 h APF and

examined at 90 h APF. n, scored clone number.

(F) Representative images and radial histogram of the quantification of the division angles at metaphase of pISC and EMC in *esg*$^{ts}$>*mira RNAi* pupal midgut. The inserts show Pros (red) and α-tubulin (white).

(G) Notch signaling reporter *NRE-lacZ* is present in ISCP (Pros$^{-}$) at 43 h APF at 30°C in *esg*$^{ts}$>*GFP* pupal midgut, and ISCP-1 (Pros$^{-}$) at 43 h APF at 30°C, and ISCP-2 (Pros$^{-}$) at 47 h APF at 30°C in *esg*$^{ts}$>*mira RNAi* pupal midgut.

(H) Quantification of the intensity of *NRE-lacZ* staining in (G). The number of measured samples are indicated in the column. Error bar, SD.

(I) Genetic interactions between Notch knockdown and mira knockdown. Pupal midguts were dissected at 66 h APF. Knockdown *mira* and Notch together showed the Notch knockdown phenotype.

(J) Statistics of Pros$^{+}$/*esg*$^{+}$ ratio referred to (I). The ratio was calculated by counting cells in a 20X image of posterior pupal midgut. Number of counted images was indicated in the column. Error bar, SD.

Scale bars, 5 μm.

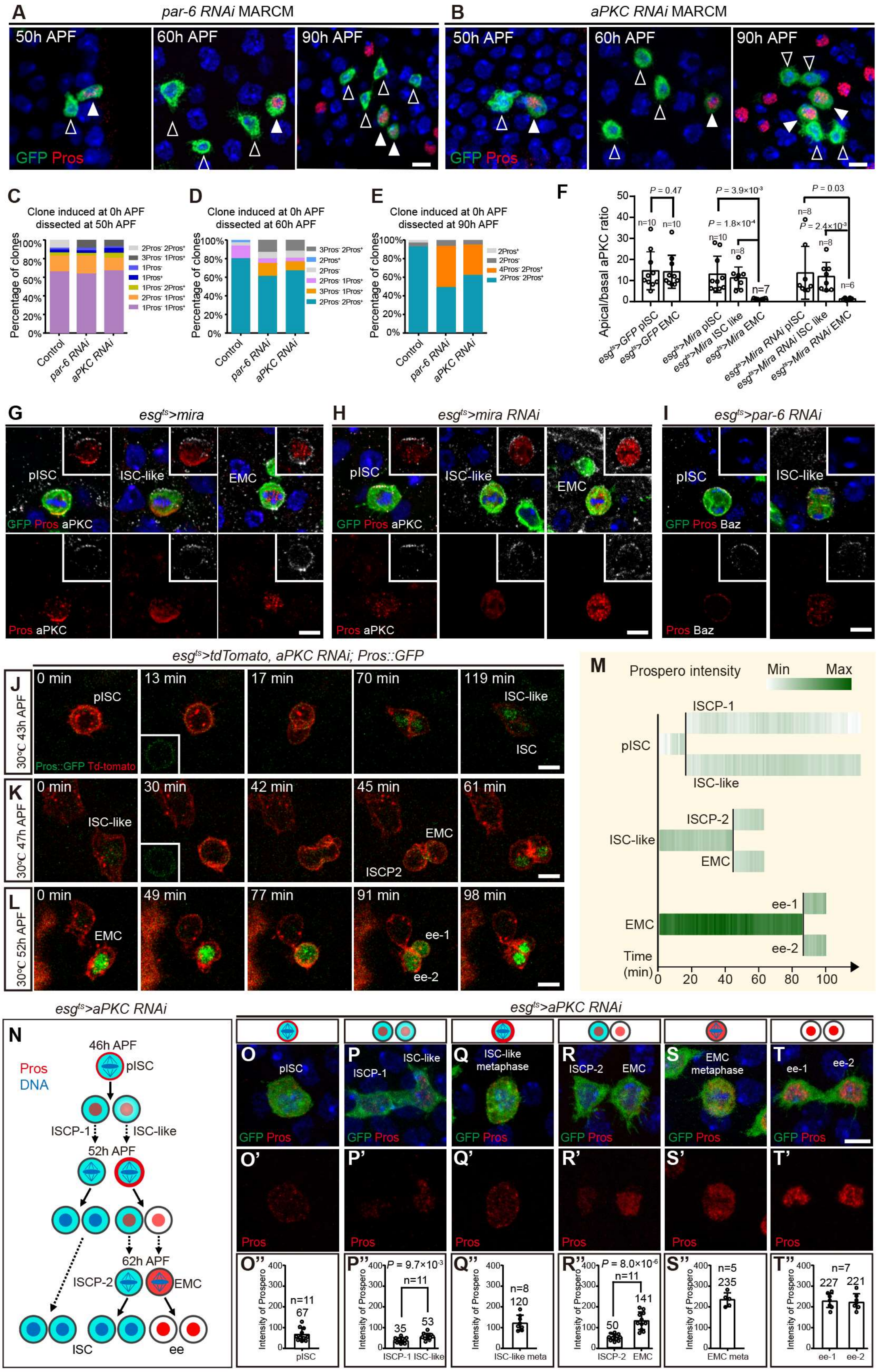


**Supplementary Figure 5. Baz:Pros ratio determines daughter cell fate in *par-6* or *aPKC* mutant pISC lineage.**

(A and B) Representative images of *par-6 RNAi* (A) and *aPKC RNAi* (B) MARCM clones induced at 0 h APF and examined at 50 h, 60 h, and 90 h APF. MARCM clone contained three $Pros^-$ cells (empty arrowhead) and one $Pros^+$ cell (white arrowhead) at 60 h APF.

(C to E) Percentage of clones in control and *par-6 RNAi*, *aPKC RNAi* MARCM clones induced at 0 h APF and examined at 50 h (C), 60 h (D), and 90 h APF (E). Clones were counted at 50 h APF: n=59 in *control*, n=58 in *par-6 RNAi*, and n=36 in *aPKC RNAi*; at 60 h APF they were n=82 in control, n=52 in *par-6 RNAi*, and n=101 in *aPKC RNAi*; at 90 h APF they were n=72 in control, n=61 in *par-6 RNAi*, and n=63 in *aPKC RNAi*.

(F) Apical/basal aPKC intensity ratio at metaphase of pISC and EMC in *esg$^{ts}$>GFP* midgut, and at metaphase of pISC, ISCP and EMC in *esg$^{ts}$>mira* and *esg$^{ts}$>mira RNAi* midgut. The dividing line between apical and basal is the equatorial plate where the chromosomes are arranged at the metaphase phase of the dividing cell. n, scored cells. Error bar, SD.

(G and H) Crescent of aPKC apically localizes during metaphase of dividing pISC and ISC-like cell in *esg$^{ts}$>mira* (G) and *esg$^{ts}$>mira RNAi* (H) pupal midguts; nevertheless, aPKC is uniformly distributed on the dividing EMC cell membrane, where Pros is localized in the cytoplasm.

(I) Pros is ubiquitously localized on the cell membrane either on pISC at anaphase or ISC-like cell at telophase/cytokinesis in *esg$^{ts}$>par-6 RNAi* pupal midgut. Baz is constantly localized on the apical cell membrane during mitotic division.

(J to L) Single frames of time-lapse movies of pISC division (J), ISC-like cell division (K), and EMC division (L) when *aPKC* was knocked down from LL3. Pros::GFP is ubiquitously localized on the cell membrane (insert in J and K) during metaphase and anaphase in *esg$^{ts}$>aPKC RNAi* pupal midgut. However, after cytokinesis of pISC mitotic division (70 min in J), Pros::GFP is increased in one of the two daughter cells (119 min in J). See also Videos S7 to S9.

(M) Diagram depicting the changes in the intensity of Pros::GFP in pISC, ISC-like, EMC, and their progenies over time in *esg$^{ts}$>aPKC RNAi* pupal midgut. The green color represents the expression of Pros according to the scale (white: low; dark green: high). Pros::GFP intensity is significantly increased in ISC-like cell than in pISC, and increased in EMC than in ISC-like cell before mitotic division.

(N) Schematic representation of the division pattern of the ISC lineage when *aPKC* was knocked down at the pupal stage.

(O to T) Representative images of Pros staining in ISC lineage after *aPKC* knockdown and the statistics of the intensity of Pros staining in the above stages. The average intensity of Pros staining (AIPS) at metaphase in the *control* pISC division is considered as 100. The value of AIPS is indicated above the error bar. The number of measured samples (n) is indicated in the chart. Error bar, SD.

Scale bars, 5 μm.

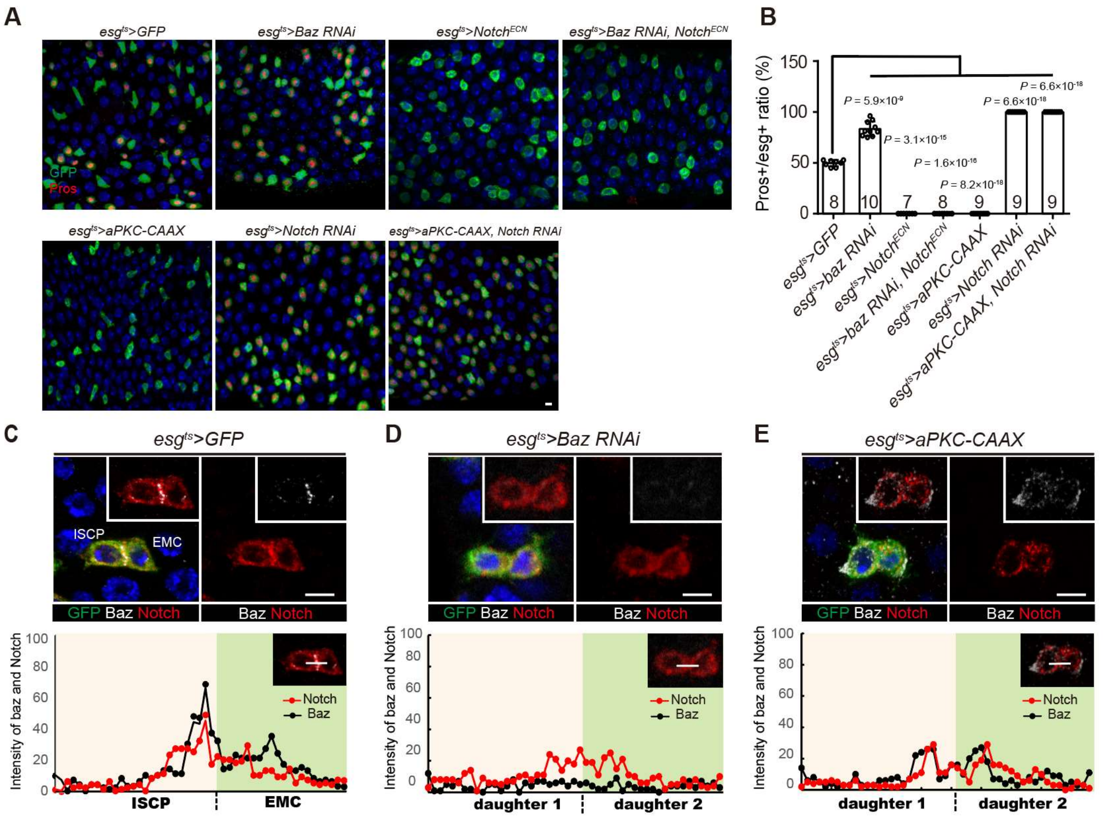


**Supplementary Figure 6. Baz promotes Notch activation through recruiting Notch receptor to the interface**

(A) Epistasis experiments of indicated conditions at at 66 h APF and 30°C. Note >*baz RNAi, Notch*$^{ECN}$ showed the same phenotype as >*Notch*$^{ECN}$, and >*aPKC-CAAX, Notch RNAi* showed the same phenotype as >*Notch RNAi*.

(B) Statistics of the ratio of Pros$^{+}$ cell/*esg*$^{+}$ cell in *control* (*esg*$^{ts}$*>GFP*) and indicated conditions of pupal midguts at 66 h APF and 30°C. All the pISC lineage cells (including Pros$^{+}$ cells) are *esg*$^{+}$ cells at 66 h APF and 30 °C. The ratio was calculated by counting all the Pros$^{+}$ cells and *esg*$^{+}$ cells in one region of interest (ROI). The number of measured ROIs (n) is indicated in the column. Error bars, SD.

(C to E) Baz and Notch receptor staining in post-dividing daughters in *esg*$^{ts}$*>GFP* (C), *esg*$^{ts}$*>baz RNAi* (D) and *esg*$^{ts}$*>aPKC-CAAX* (E) pupal midgut. Baz and Notch fluorescence distributions crossed the interface (white bar in the upper-right corner) were measured below.

Scale bars, 5 μm.

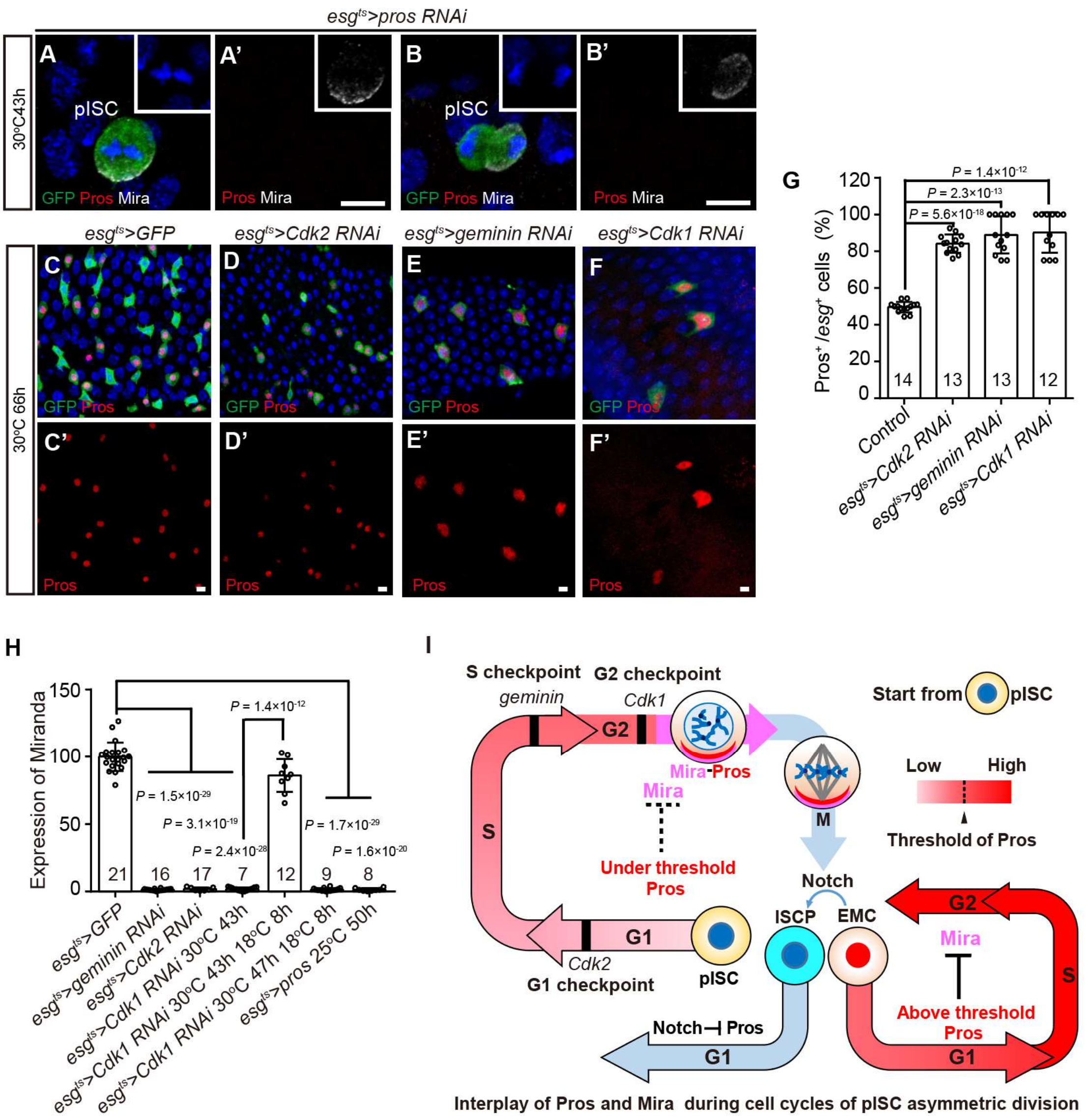


**Supplementary Figure 7. G1 Pros expression precedes G2 Mira expression and Pros inhibits the transcription of Mira after reaching the threshold.**

(A and B) High Mira accumulation on the basal side of pISC cell membrane at metaphase (A) and on the basal side of pISC daughter cell membrane at telophase (B) in *esg*$^{ts}$>*pros RNAi* intestines, while no Pros staining is found at 43 h APF at 30°C. The inserts in (A) and (B) show DAPI staining of the nucleus (white), indicating the stage of the cell cycle. The inserts in A' and B' show Mira staining.

(C to F) Representative images of *esg*$^{ts}$ driven GFP and Pros expression in the control (C) and genotype indicated pupal midguts (D-F) at 66 h APF and 30°C. Polyploid nuclei are present in *geminin RNAi* and *Cdk1 RNAi* pupal midguts, indicating the S cell cycle arrest.

(G) Statistics of the ratio of Pros$^{+}$ cell/*esg*$^{+}$ cell in *control* (*esg*$^{ts}$>*GFP*) and in the indicated conditions of pupal midguts at 66 h APF and 30°C. All the pISC lineage cells (including Pros$^{+}$ cells) are *esg*$^{+}$ cells at 66 h APF and 30 °C. The ratio was calculated by counting all the Pros$^{+}$ cells and *esg*$^{+}$ cells in one region of Interest (ROI). The number of measured ROIs (n) is indicated in the chart. Error bars indicate SD.

(H) Quantification of the intensity of Mira staining under indicated genetic conditions. The number of measured samples are indicated in the column. Error bars indicate SD.

(I) Schematic representation showing the interplay of Pros and Mira during cell cycles of pISC asymmetric

division. The red color represents the expression of Pros according to the scale (pale red: low; strong red: high). The dash line in the up-right indicated the threshold of Pros sufficient to inhibit Mira expression. The pink color represents Mira expression. G1 Pros expression precedes G2 Mira expression and Pros inhibits the transcription of Mira after reaching the threshold. During mitosis in pISC, Pros that do not reach threshold are segregated by Mira to the basement membrane and reach threshold in EMC to repress mira expression.

Scale bars, 5 μm.
